# Cross-Dialectal Novel Word Learning and Borrowing

**DOI:** 10.3389/fpsyg.2021.734527

**Published:** 2021-09-30

**Authors:** Junru Wu, Wei Zheng, Mengru Han, Niels O. Schiller

**Affiliations:** ^1^Laboratory of Language Cognition and Evolution, Department of Chinese Language and Literature, East China Normal University, Shanghai, China; ^2^Leiden University Centre for Linguistics, Leiden, Netherlands; ^3^Leiden Institute for Brain and Cognition, Leiden, Netherlands

**Keywords:** dialect, lexical borrowing, word learning, lexical processing, bilingualism

## Abstract

The objective of this paper was to study the cognitive processes underlying cross-dialectal novel word borrowing and loanword establishment in a Standard-Chinese-to-Shanghainese (SC-SH) auditory lexical learning and borrowing experiment. To investigate these underlying cognitive processes, SC-SH bi-dialectals were compared with SC monolectals as well as bi-dialectals of SC and other Chinese dialects (OD) to investigate the influence of short-term and long-term linguistic experience. Both comprehension and production borrowings were tested. This study found that early and proficient bi-dialectism, even if it is not directly related to the recipient dialect of lexical borrowing, has a protective effect on the ability of cross-dialectal lexical borrowing in early adulthood. Bi-dialectals tend to add separate lexical representations for incidentally encountered dialectal variants, while monolectals tend to assimilate dialectal variants to standard forms. Bi-dialectals, but not monolectals, use etymologically related morphemes between the source and recipient dialects to create nonce-borrowing compounds. Dialectal variability facilitates lexical borrowing *via* enriching instead of increasing the short-term lexical experience of learners. The long-term bi-dialectal experience of individuals, as well as their short-term exposure to each specific loanword, may collectively shape the route of lexical evolution of co-evolving linguistic varieties.

## Introduction

Few languages in the world come with no loanwords. Loanwords are very common if lexical borrowing across co-evolving dialects is taken into consideration. Lexical borrowing across dialects prevails with practical significance. For instance, new words, e.g., *computer* in the 1980s, since they are introduced to a new linguistic community, usually primarily enter the more prestigious dialect and then spread from such a dialect (the source dialect) to other dialects (the recipient dialects). This ongoing historical process deeply shapes the current appearance of co-evolving dialects, as well as co-evolving languages.

Borrowing is a term usually used in classic linguistic studies of language evolution. However, language evolution receives profound collective influences from individual behaviors (Trudgill, 1986; Fitch, 2007). Specifically, lexical borrowing is realized through the cross-linguistic behaviors of bilinguals and monolinguals, which is known as a common way that individuals modify their speech (*accommodate* lexical forms) in order to match those of their interlocuter (Giles et al., 1973; Trudgill, 1986)^1^.

Since the collective behaviors of individuals are considered for the historical course of lexical borrowing, it is reasonable to assume that the way borrowing takes place is influenced by the cognitive costs it charges from individuals. Regarding what makes it difficult to borrow words, numerous linguistic debates have arisen in the past few centuries, e.g., on the existence of *substratum interference* in the linguistic society (as reviewed by Thomason and Kaufman, 1991) and on lexical features (as reviewed by Wang and Wang, 2004). However, it remains unclear in many aspects how this borrowing is related to the cognitive processing of words and influenced by the experiences of individuals.

For instance, bi-dialectals are the group that most frequently is involved in lexical borrowing while interdialectal lexical borrowing gives rise to *interdialectal forms*, as noted in the study by Trudgill (1986), which deeply shapes the results of dialectal co-evolution. However, little is known about when and why bi-dialectals would find a source form difficult to borrow. Therefore, the current research offered a *psycholinguistic* approach to understand this problem.

To our knowledge, although some recent studies had investigated interdialectal lexical adaptation, e.g., Swerts et al. (2021), very few experimental studies had directly tapped into the issue of lexical borrowing in the context of bi-dialectism. Hence, before moving on to the psycholinguistic backgrounds of the current research, we introduced one sociolinguistic study which has critically discussed individual behaviors in lexical borrowing.

### From Nonce Borrowing to Established Loanwords

Typical “loanwords” are widely accepted as words that recur relatively frequently. These words are widely used and have achieved a certain level of acceptance in the recipient linguistic community (Mackey, 1970; Poplack and Sankoff, 1984). These features distinguish established borrowing from single-word code-switching. However, the research of Poplack et al. (1988) studied Canadian English-French/French-English bilinguals from highly bilingual communities and have proven the existence of *nonce borrowing*, known as loans which are adapted from source-language words and used incidentally in recipient languages. These borrowings have two important features. First, they are incidental and realized with inconsistent pronunciations, in contrast to established borrowings, which usually have established loan forms. Second, they usually involve phonological and morphological adaptation, in contrast to single-word code-switching, which maintains the source forms in the recipient context.

Although most nonce loanwords are short-lived, we presumed that nonce borrowing in language evolution may be the predecessor of established borrowing. One type of supportive evidence may be the one-to-many mapping in the early stages of lexical borrowing which has been documented in the literature. For instance, the Sanskrit word “Buddha” yielded at least four Chinese phonological loan forms between 220 and 589AC (Ji, 1948), namely, 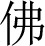 ^*^bṷot^33^, 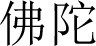 ^*^bṷot^33^dα^35^, 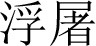 ^*^biu^33^dṷo^33^, 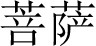 ^*^bṷo^33^sαt^3^. If we had taken into consideration all the documented pronunciations of these characters in Middle Chinese rime books, the same Sanskrit source word “Buddha^2^” would be associated with even more Chinese loan pronunciations in that historical period, when Sanskrit words were introduced by bilingual monks into Chinese in large quantities with the spread of Buddhism. Similar lexical variation was reported in the study by Poplack et al. (1988) for contemporary nonce-borrowing in the French-English bilingual society, only that most of those nonce loan forms as documented did not get established in that linguistic society. Similarly, studies tracking the history of modern loan words have also shown a pattern that, at the start of a language contact case, with the introduction of new concepts, many candidates of loan forms come to existence, frequently with competing lexical forms associated with the same source forms. However, after the beginning stage of contact, usually only one or two loan forms are kept for each loanword in the linguistic community. For instance, until 2009, 56% of Shanghainese (SH) loan forms of foreign origin that had been documented in 1945 have been replaced and many of the remained have taken altered pronunciations or written with different Chinese characters (You, 2016). However, psycholinguistic mechanisms involved in individuals who made the collective unconscious decision in loan form establishment are still worth investigating.

When loanwords are getting established, the collective influence from individuals probably starts taking effect when individuals first hear and/or produce the loan forms with meaning. Hence, studying the cognitive processes involved in the establishment of nonce loanwords may provide a new perspective that helps one to understand the cognitive mechanism involved in the co-evolution of closely related language varieties.

To study this collective individual course of loan establishment, the current experimental study operationalized the research question by comparing *new nonce loanwords* and *incidentally established loanwords* in lexical recognition and production.

Specifically, *source novel words* were created by combining novel meanings with novel forms in the source language to exclude the influence of previous lexico-specific knowledge. Then, recipient *nonce loanwords* were created for these source words. To control for the way and extent the loanwords differ from the source words, these loanwords were all designed with morphological and phonological adaptations.

1. After the participants acquired the source novel words with meaning, some of the corresponding loanwords are auditorily presented to the participants for them to figure out the meaning. In this process, the participants need to transfer their previous and recently acquired lexical knowledge, i.e., about the source novel word, to recognize the novel loans. This is the *comprehension borrowing* of novel nonce loanwords, which mimics the very beginning of loan receiving in a linguistic community. However, note that comprehension borrowing may be more possible between dialects because dialects have a relatively high degree of mutual intelligibility (for example, see Wang and Van Heuven, 2015 for an investigation on the mutual intelligibility across Chinese dialects), while between remote languages there would be only the semantic and phonological similarities of the source and recipient forms to rely on.

2. Having experienced such auditory exposure to these loans, the participants had briefly established the lexical representations of these loans in their memory. Therefore, these loans had become *incidentally established loanwords*. When these participants were asked to name images (which represent the lexical meaning) with the *incidentally established loanwords*, this is the *production borrowing of incidentally-established loanwords*, which mimics the first usage of bi-dialectal individuals of a newly acquired loanword in the bidialectal community.

3. We prepared other novel loanwords that were not presented to the participants in the comprehension borrowing test, and the participants only learned the corresponding source forms with meaning. Afterwards, these novel loanwords remained secret in the recipient dialect. However, the participants were still asked to name the images corresponding to these loanwords in the recipient dialect. In this way, they had no other option but to create these nonce loanwords by themselves. To achieve this goal, they needed to actively transfer their lexical-specific knowledge from the source dialect, as well as their previous knowledge on the phonological and morphological relations between the two dialects (if available). This is the *production borrowing of nonce loanwords*, which mimics the first creative usage of bi-dialectal individuals of nonce loanwords in their bi-dialectal community (*lexical creation*; Weinreich, 1953).

By comparing the production borrowing of nonce loanwords and that of incidentally established loanwords, we could tap into the cognitive processes involved in the very beginning of loanword establishment. Also, we could study the cognitive process underlying the comprehension and production of nonce loanwords.

Nonce borrowing appears more frequently in highly bilingual linguistic communities (Poplack et al., 1988), which suggests that the cognitive processes involved may be influenced by community members' long-term bilingual experience. Hence, we further investigated how socio-linguistic background may influence the cognitive performance of individuals in the establishment of loanwords. This is related to previous cognitive findings on word learning and bilingual lexical processing, which are reviewed as follows.

### Age Effects in Novel Word Learning and Mental Establishment of Loanwords

**Word learning** is influenced by many factors, of which the factor age was the focus of the current research on lexical borrowing. While many studies have shown age-related deterioration of phonological and syntactic learning abilities (Lenneberg et al., 1967; Werker and Hensch, 2015; Reh et al., 2021), contradicting findings in studies on the vocabulary development of deaf individuals have triggered a long-lasting debate about the existence of the sensitive period for word learning (Newport et al., 2001; Lederberg and Spencer, 2005; Connor et al., 2006).

The influence of age on word learning becomes more complicated when bilingualism is taken into consideration. On the one hand, the early age of L2 acquisition may create a more integrated bilingual mental lexicon (Sabourin et al., 2014; Cardimona et al., 2016). On the other hand, although non-dialectal bilinguals as compared with monolinguals usually show lexical disadvantages (as reviewed by Bialystok, 2009), such a disadvantage is largely absent by early and proficient bi-dialectals (Wu, 2015, pp. 143–188; Wu et al., 2019), who are special in the sense that they are highly experienced with cross-dialectal lexical learning and lexical borrowing. Therefore, it is reasonable to ask whether bi-dialectal exposures would delay the deterioration of word-learning ability.

Naturally, this question is raised under the presumption that there is an age-related deterioration of word-learning ability by monolectals, which was verified in the current research. Then, if it was found that bi-dialectals maintain better word-learning abilities until a later age, a follow-up question would be whether this advantage can further benefit their lexical borrowing. In order to focus on the influence and interaction of bi-dialectism and age, this study controlled all the other known predictors for word learning (Kaczer et al., 2018), including the availability of semantic information (for examples, see Gaskell and Dumay, 2003; Qiao and Forster, 2013; Li and Xu, 2021), referential familiarity (Barcroft and Sunderman, 2008; Kaushanskaya et al., 2013), contexts (e.g., Mestres-Missé et al., 2008; Lindsay and Gaskell, 2013), semantic clustering (e.g., Tinkham, 1997; Finkbeiner and Nicol, 2003; Erten and Tekin, 2008), form variability (Lively et al., 1993; Keuleers et al., 2007; Kriengwatana et al., 2014), and tasks (for e.g., see Forster, 1985; Jiang and Forster, 2001; Witzel and Forster, 2012; Qiao and Forster, 2017). Within all the known semantic predictors for word learning, merely one factor was considered in this research, namely the semantic concreteness of morphemes that form the novel words. This semantic factor was considered because it covaries with a morphological probability of the morphemes in the recipient dialect (see a further explanation later).

### Bi-Dialectal Particularities in Cross-Dialectal Lexical Borrowing

**One aim of the current study was to test for potential cognitive particularities of bi-dialectals in interdialectal lexical borrowing**. Thus, we compared bi-dialectals against monolectals of comparable linguistic backgrounds.

As few studies directly compared bi-dialectals and monolectals in lexical processing, here we primarily introduced known cognitive differences on the cross-linguistic lexical processing between bilinguals and monolinguals. Early and proficient bilinguals (Antoniou et al., 2012; Wu et al., 2017; De Leeuw and Celata, 2019; Wig and García-Sierra, 2021) seem to be more adaptive than monolinguals (for e.g., see Best and Strange, 1992, Perceptual Assimilation Model, PAM) in speech perception. Also, compared with L2 learners, early and proficient bilinguals are more likely to maintain separate instead of joined lexical representations for etymologically-related translation equivalents (ETEs), i.e., word pairs/sets that have common origins, refer to the same concepts, and are similar in sound, which is either cognates inherited from a common ancestor language or loans borrowed across languages. These ETEs are cognitively processed differently as compared with language-specific words (e.g., Sumner and Samuel, 2009; Dijkstra et al., 2010; Mulder et al., 2015; Larraza and Best, 2018; Wu et al., 2019).

Hence, we hypothesized that when carrying out cross-dialectal lexical borrowing, early and proficient bi-dialectals might be biased toward adopting the adaptive perceptual mechanisms and create separate lexical representations across dialects, while monolectals might be biased toward perceptual assimilation and joined lexical representations. This claim probably also applies to production borrowing, considering the studies of Poplack et al. (1988) and Ernestus and Baayen (2003) as mentioned above.

Interestingly related to these findings, previous phonological studies on lexical borrowing have also distinguished phonological and phonetic adaptions (for e.g., see Kang, 2010) as well as adaption vs. direct surface adoption (Aktürk-Drake, 2014). These studies also suggested that choices during loan adaptation may be modulated by both perceptual factors and the familiarity of individuals with the source language, e.g., foreign vs. near-native.

To test for the alternative mechanisms of bi-dialectals and monolectals when it comes to lexical borrowing, the current study adopted similarity-related interference as a probe for the emergence of lexical representation (Marian et al., 2008; Dijkstra et al., 2010; Wu et al., 2019) and used novel ETE forms of recently learned novel words to control for prior lexical-specific experience. This approach assumed that only separate lexical representations could interfere with each other. Participants who primarily assimilated loan forms to source lexical representations would primarily show similarity-related facilitation, while participants who created new lexical representations for the loan forms would show interfering or non-linear similarity effects. Also taking into consideration the above-mentioned different biases of monolinguals vs. bilinguals, we expected monolectals to show similarity-based facilitation and bi-dialectals to show interfering or non-linear effects of similarity.

Following the question on the particularities of bi-dialectals, another question requires further investigation, i.e., whether previously observed bilingual lexical effects resulted from the additional lexical experience of bilinguals with specific languages, or contrasts of cognitive features by bilinguals vs. monolinguals, or both.

The current study tapped into this question by exposing monolectals and two groups of bi-dialectals to the same source and recipient dialects. The long-term linguistic experience of bi-dialectals differed in a way that one group of bi-dialectals (Standard-Chinese-Shanghainese; SC-SH bi-dialectals) were familiar with the recipient dialect (Shanghainese; SH) and the other bi-dialectals (Standard-Chinese-Other-Dialect; SC-OD bi-dialectals) were not, as shown in Table 1. We predicted that effects specific to the experience with the recipient-dialect should only be found by the former group, while non-specific bi-dialectal effects should be found by both groups of bi-dialectals. Non-specific bi-dialectal exposure may change the way bi-dialectals of the other Chinese dialects integrate or create new lexical representations, and bi-dialectals may be able to apply this operational knowledge to unfamiliar dialects. Moreover, if non-specific long-term bi-dialectism takes effect, we expected to find evidence for the other bi-dialectals in the recipient dialect that they do not speak (i.e., SC-OD bi-dialectals in SH) also to create separate lexical representations for source and loan forms.

**Table 1:** Different types of long-term linguistic experience (header column) and their predicted influences on the effects of dialectal backgrounds (header row).

	**Dial. background**
**Long-term ling. Experience**	**SC monolectals**	**SC-OD bi-dialectals**	**SC-SH bi-dialectals**
Long-term bi-dialectal exposure	No	Yes	Yes
Long-term recipient-dialect-specific experience	No	No	Yes
Lexical semantic experience to morphemes	Yes	Yes	Yes

Hence, this study compared the lexical borrowing processes of bi-dialectals and monolectals and investigated whether the differences originate from different specific experiences to the recipient dialect or from bi-dialectism in general.

### Holistic Casting versus Morpheme-Based Re-encoding in Lexical Borrowing

**Another issue to be investigated is holistic casting vs. morpheme-based re-encoding in lexical borrowing**. There have been many sociolinguistic and historical discussions regarding the hierarchy of linguistic units under contact. Chinese dialects, as analytic in typology, share many etymologically aligned morphemes, which also form many etymologically aligned compounds, can serve as an ideal test case. Actually, in the contact linguistic literature, as long as Chinese is involved, mono-syllabic morphemes are usually assumed as the primary units of borrowing (for e.g., see Wang and Lien, 1993; Xian, 2012), whereas cross-linguistic borrowing can also involve phonological adaptation of sub-syllabic units such as consonant onset, rhymes, and tones (for e.g., see Yang, 1982; Wang and Lien, 1993; Wang, 2005), which can be attributed to word-wise phonological adaptation. Although the effects of word boundaries are less studied in those works, in studied cases, parallel loan forms that can be respectively attributed to morpheme-based and whole-word-based are rather frequent, especially between originally unrelated but co-evolving languages. For instance, in the case of Korean language which has been borrowing Chinese morphemes and words throughout the past two thousand years, the Chinese word 上海, ‘Shanghai' (up + sea), has two parallel loan forms, which are “holistically casted” 
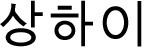
, /sang.ha.i/, and “morpheme-based” 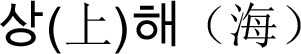/sang.he/ (Yu and Wu, 2021). So far, little is known about the cognitive base for the emergence of such lexical variance.

In other languages, it is also the case that previous experimental studies have supported both the phonological (for e.g., see Peperkamp et al., 2008; Aktürk-Drake, 2014) and morphological adaptations during nonce borrowing. For instance, the study of Ernestus and Baayen (2003) found that lexical phonotactics of recipient-language morphemes would guide the selection of phonemes in creating nonce loanwords. Hence, both phonological adaptation and morpheme-based lexical creation processes may be involved in the creation of nonce loanwords.

Moreover, cognitive studies on word-learning have also shown that learners shift their attention from separated constituents to the whole words when they learn compound words (Kaczer et al., 2015). These findings inspired the current research to investigate the activation of morphological constituents and the emergence of whole-word representations during lexical borrowing.

Thus, the question lies on whether and when individuals would cast the source form holistically into a new form in the recipient language or re-encode etymologically related morphemes between the two linguistic varieties.

To investigate this question, the current research used novel compounds made of existing morphemes as learning targets and adopted morphological probability as a probe predictor for the activation of constituents. While the morpheme frequencies of the novel compounds in the recipient dialect were manipulated, their morpheme frequencies in the source dialect were controlled. In this way, the morphological probability of the novel compounds in the recipient dialect was implicitly manipulated. For instance, the Chinese morphemes 脖 “neck” and 拖 “drag” have similar frequencies in SC as /bo35/ and /thuo55/. However, frequencies of their counterparts differ greatly in SH: the character 脖/bo23/“neck” is much less frequent than the character 拖 /thu55/ “drag” because SH primarily uses another morpheme, éćĹ /tɕiŋ55/, for the concept “neck.”

With this manipulation, the appearance of morphological-probability effects during lexical borrowing would indicate the activation of morphological constituents in the recipient dialect and hence would imply morpheme-based re-encoding. Specifically, if it was observed that a loan compound that was morphologically less probable in the recipient dialect, e.g., ^*^脖 澡 /bo11dzho23/ which contains the SH less frequent脖 /bo23/, “neck,” triggered significantly larger processing cost than a morphologically-more-probable loan compound, e.g., ^*^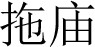/thu55mi*O*21/ which contains the SH more frequent拖 /thu55/, “drag.” This would indicate that morphemes in the recipient dialect are activated. Moreover, considering the above-mentioned potential differences between monolectals and bi-dialectals with respect to lexical-borrowing mechanisms, we predicted that bi-dialectals but not monolectals would be sensitive to morphological probability in the recipient dialect during both comprehension and production borrowing.

Note that the morphological probability of the ETE loanwords in SH covaries with semantic concreteness of compound constituents. This is because the recipient dialect SH is a less-prestigious Chinese dialect and less-prestigious dialects tend to borrow more literary words from national standards (standard varieties, a type of “high variety” as termed by Ferguson, 1959) while keeping their own everyday words^3^. Abstract words, e.g., union, are usually more literary than concrete words, e.g., nose, in their meaning (Dixon, 1997). As a result, with the morphological probability of novel words controlled in the source dialect SC, higher morphological probability in SH (a non-standard variety) is related to higher concreteness.

However, the current design could distinguish probability and concreteness effects. Since SC monolectals have no prior knowledge of SH morphological probabilities, it was reasonable to predict that real effects of Shanghainese morphemes were only to be found by SC-SH bi-dialectals, and perhaps also by SC-OD bi-dialectals (which would be mediated by the probability of SC-originated morphemes in these non-prestigious Chinese dialects), but not by the SC monolectals. In contrast, since ETE morphemes were semantically aligned, if an observed effect of Shanghainese morphological probability is mediated by concreteness, the effect should be found consistently across groups.

Hence, this study manipulated the morphological probability of novel compounds in the recipient dialect to investigate whether and at which stage of lexical borrowing the constituents of loan compounds emerge in the mental lexicon of the borrowers.

### Conflict Resolution and Inference in Cross-Dialectal Lexical Borrowing

**In the context of cross-dialectal lexical borrowing, conflict resolution and inference were also investigated in this study**. Bilinguals are known to show cognitive advantages relative to monolinguals in conflict resolution and attentional control (as reviewed by Bialystok, 2009). However, little is known about whether bilinguals and monolinguals differ in how they infer lexical meanings from the exterior context. Only that the *mutual exclusivity principle* (Markman, 1992) may work differently by monolingual and bilingual children in some situations (Davidson and Tell, 2005; Kalashnikova et al., 2015), wherein the older bilingual children are more able to accept lexical overlap, but they are not always different (e.g., see Frank and Poulin-Dubois, 2002).

The current study manipulated the referential context in the comprehension borrowing session to compare the practice of bi-dialectals and monolectals in resolving referential ambiguity during comprehension borrowing. In the preconditioning learning phase of novel SC words, we included some images with no explicit names given but accompanied with white noise. Then in the comprehension borrowing test, trials with auditory words and trials with white noise were included.

First, to test for the effects of conflict resolution, the word trials compared a high-competition context (target image accompanied with a distractor image which had been associated with a word) with a low-competition context (with a distractor image which had been associated with noise). Second, to test for the potential difference between bi-dialectals and monolectals in inferencing lexical references with the *mutual exclusivity principle*, the noise trials compared an inference-possible context (with a distractor image associated with a word) with an inference-impossible context (with a distractor image also associated with noise).

In sum, the current research adopted a modified experimental neologism paradigm in a SC-SH auditory lexical borrowing experiment by monolectals and bi-dialectals. Both comprehension and production borrowings were tested to investigate the roles of long-term and short-term linguistic experience on lexical borrowing.

## Materials and Methods

### Participants

**Figure 1:**
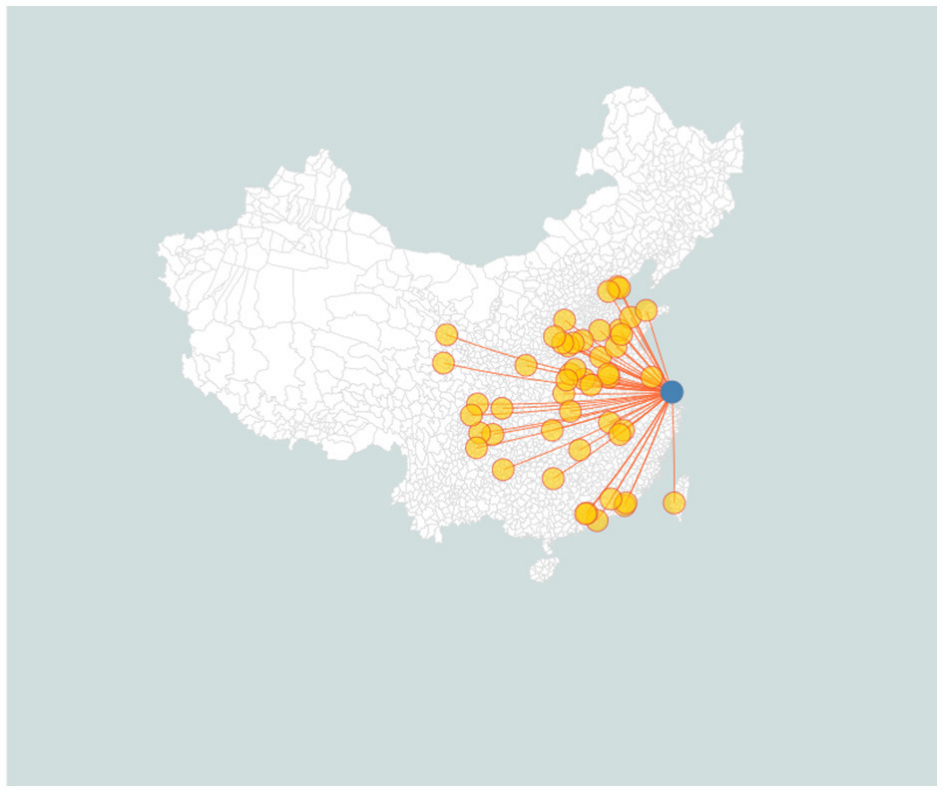
Locations of the dialects of SC-OD bi-dialectal (yellow-filled circles) and Shanghai (the blue solid circle) on a map of China. The empty areas in the map are either deserts with little population or where non-Chinese languages are primarily spoken besides SC.

Three groups of participants participated in this experiment in exchange for payment. (1) Native SC monolectals who only speak SC natively and no other Chinese dialects were 28 in total, 4 men and 24 women, age 17–34, *M* = 21.35, *SD* = 4.04, self-rated SC proficiency (on a 0–10 scale) 5~10, *M* = 8.89, *SD* = 1.22. The other two groups were bi-dialectals. (2) Standard-Chinese-Shanghainese bi-dialectals who speak both the source dialect SC and the recipient dialect SH natively were 37 in total, 13 men and 24 women, age 18~47, *M* = 24.27, *SD* = 7.13, self-rated SC proficiency (on a 0–10 scale) 4~10, *M* = 8.65, *SD* = 1.37, SH proficiency 5~10, *M* = 8, *SD* = 1. (3) Standard-Chinese-Other-Dialects bi-dialectals who speak the source dialect SC and a non-Wu^4^ Chinese dialect natively were 34 in total, 5 men and 29 women, age 18~30, *M* = 22.41, *SD* = 2.33, self-rated SC proficiency (on a 0–10 scale) 6~10, *M* = 8.65, *SD* = 1.22, self-rated OD proficiency 2~10, *M* = 7.63, *SD* = 1.97. The locations of the dialects of SC-OD bi-dialectals are shown on a map of China in Figure 1.

All participants acquired their literacy in SC and learned some English at school. A few participants from each group also had some knowledge of other foreign languages. Due to the limitation of recruiting conditions, all three groups had a long-tail age distribution, skewed to the left (younger side).

### Design and Procedure

**Figure 2:**
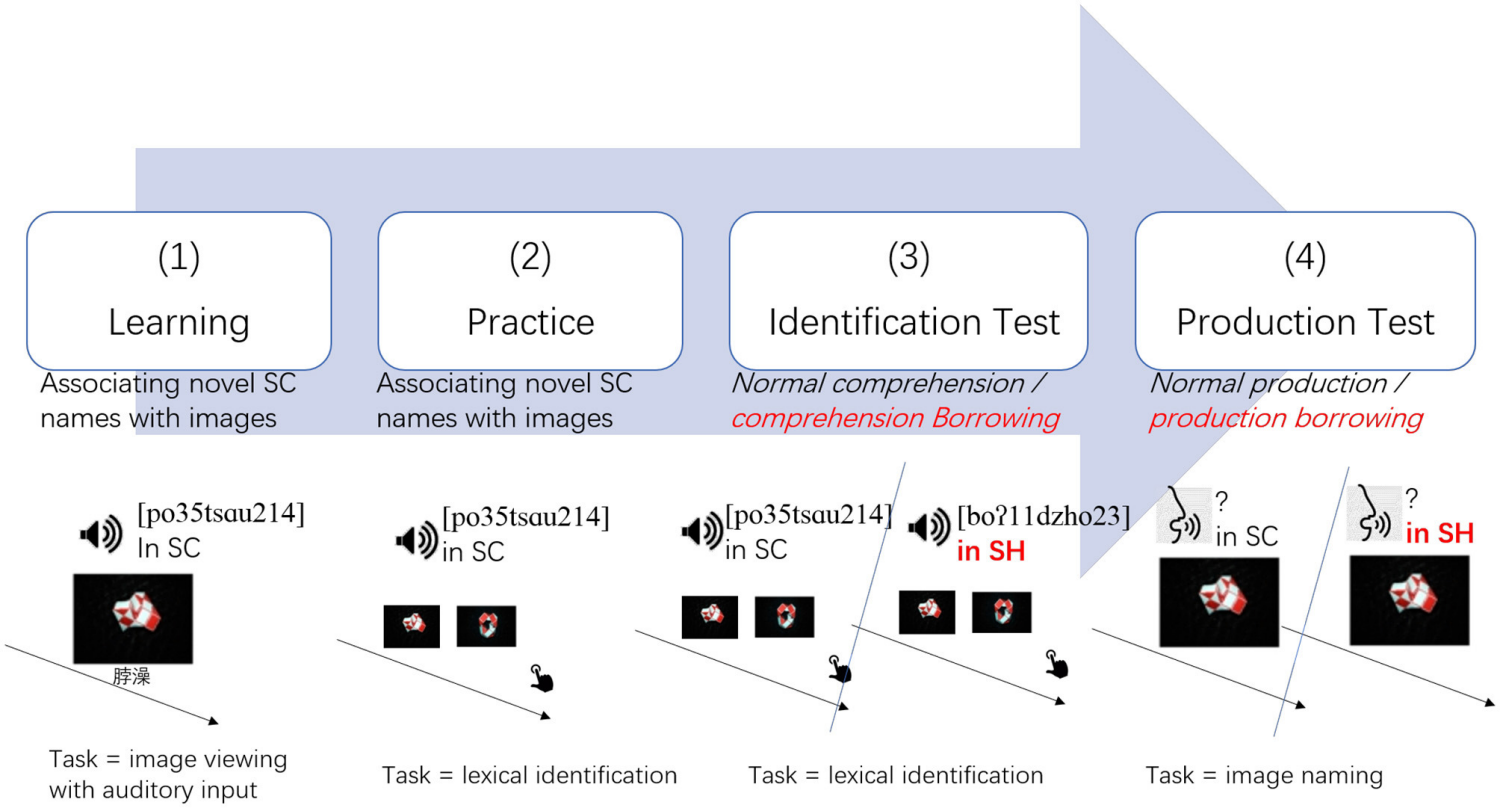
Experiment Procedure, including four phases: (1) Learning, (2) Practice, (3) Identification Test, (4) Production Test.

This study adopted an experimental neologism paradigm. As shown in Figure 2, first, in a preconditioning phase, participants learned to associate aurally given names (nonce compounds made of existing Chinese morphemes) with images of novel shapes in SC. They were trained with a “choose-one-from-two” audio-image lexical identification task in SC until their accumulative accuracy reaches 85%. For images associated with white noise in the learning phase, either choice is taken as correct in the practice phase. Afterwards, they were tested again in both SC and SH with an audio-image lexical identification task (comprehension test) and then an image-naming task (production test).

A mixed design was adopted. (1) Long-term linguistic experience was manipulated between participant groups, which included *dialectal background* (*SC monolectal/SC-SH bi-dialectal/SC-OD bi-dialectal*), *age*, and *subjective proficiency in SC* of the participants. (2) Short-term linguistic experience were manipulated within groups, including the *morphological probability* of novel compounds in SH (*more/less probable*, also corresponding to *more/less concrete* meanings of the morphemes), *cross-dialectal similarity* (scaled) between source-dialect and recipient-dialect forms, and *dialect of operation* (*SC* = source dialect/*SH* = recipient dialect).

In addition, the design for the auditory identification test also manipulated exterior *context* provided by the distractor (*high/low-competition for the novel words & inference possible/impossible for the noise*). Moreover, for the production task, the factor *dialect of operation* was combined with *the exposure of the participants to target words prior to the naming task*, which formed a new factor lexical-specific *learning experience (bi-dialectal exposure and tested in SC/exposure only in SC and tested in SC/bi-dialectal exposure and tested in SH/ exposure only in SC and tested in SH)*.

As shown in Table 2, during the learning phase each participant was asked to remember four name-image associations in SC. Second, during the identification test after practice, participants were required to identify an aurally presented word by choosing from two given images in each trial. They identified two associations in SC and the other two in SH (comprehension borrowing). Each participant identified each association twice in each dialect, first with a high-competition context, and then with a low-competition context. Third, in the picture-naming test, participants named one of the two associations which they just identified in SC again in SC and the other in SH. Similarly, they also named one of the two associations which they just identified in SH and the other in SC. In the naming task, they were first tested in SC and then in SH. Separate recordings were collected from the onsets of visual stimuli until the beginning of the next visual stimuli.

**Table 2:** Experiment procedure (normal fonts) and the corresponding theoretical implications (bold fonts).

**Phases**	**(1) Learning**	**(2) Practice**	**(3) Identification test**		**(4) Production test**		**Theoretical implications**
**Dialect**						
Cond. 1	SC	SC	–	SH	SC	–	Backward influence (from recipient to source dialects)
Cond. 2	SC	SC	SC	–	SC	–	Source-dialect baseline
Cond. 3	SC	SC	–	SH	–	SH	Incidentally-established borrowing
Cond. 4	SC	SC	SC	–	–	SH	Nonce borrowing

In the learning phase, participants also learned four images without names (presented with white noises). In the identification test, these noise associations were mixed with associations with names and each association was tested twice, first in inference-possible context, and then in inference-impossible context. Participants were told that either image chosen would be treated as correct for the noise trials.

The design avoided presenting names with shared or similar morphemes to the same participant. The same eight images were given to all the participants but were associated with different names for different participants so that more names with a wide range of SH morphological probability can be tested. Each participant group was divided into sub-groups so that name-image associations can be similarly counterbalanced within each group.

After the experimental neologism experiment, all participants rated all the SC-SH name pairs on a five-point scale for the *cross-dialectal similarity* measurement.

### Stimuli

#### Novel Names

**Figure 3:**
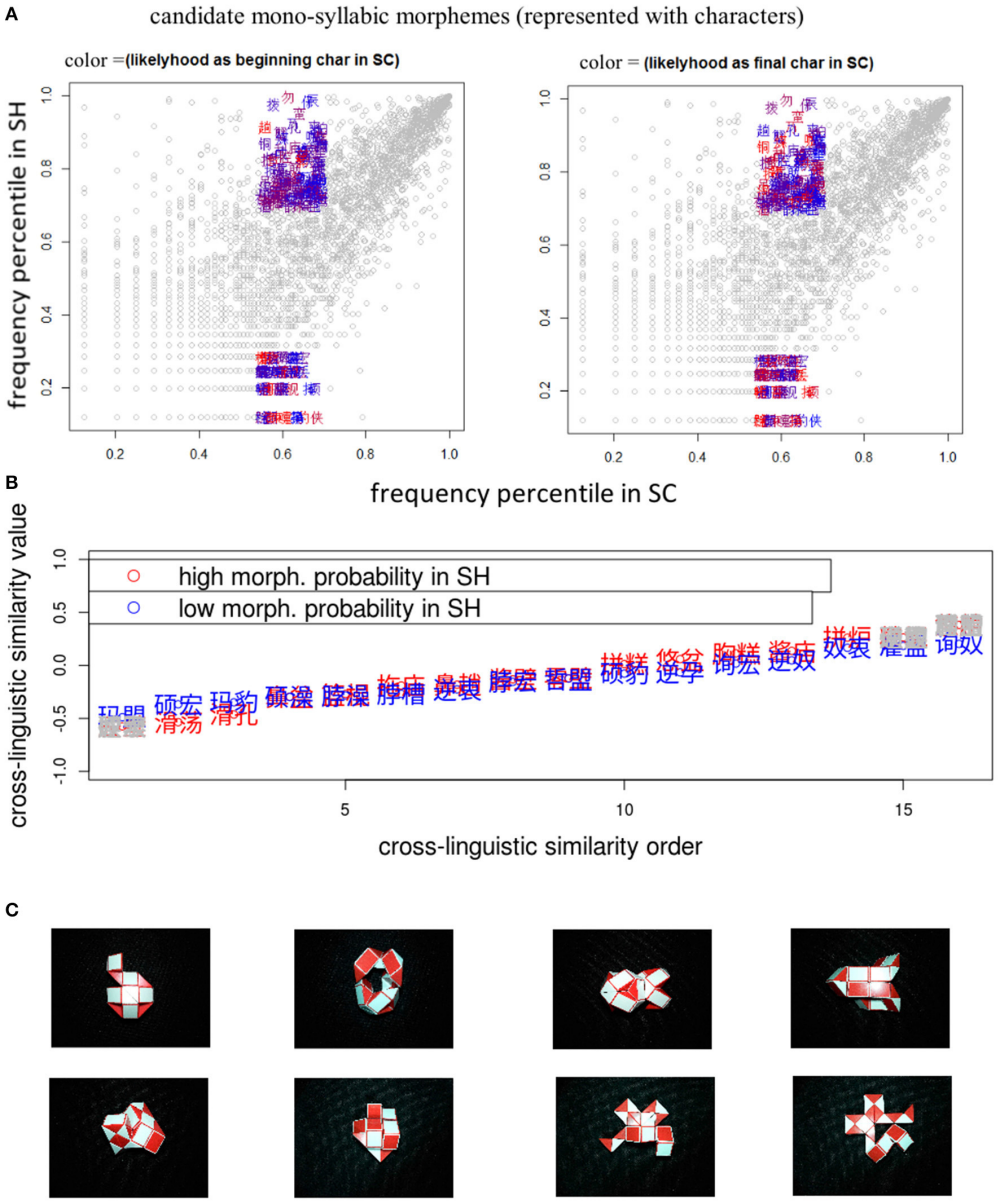
**(A)** Upper panel: scattered plots for candidate mono syllabic ETE morphemes, represented with Chinese characters, plotted with frequency percentiles in SC (along the horizontal axis) and SH (along the vertical axis), colored from blue (low) to red (high), according to the likelihood as beginning (left sub-plot) and final characters (right sub-plot). **(B)** Middle panel: scattered plots for two sets of disyllabic novel ETE pairs, represented with Chinese characters, plotted with the order (along the horizontal axis) and values (along the vertical axis) of cross-dialectal similarity, colored in blue for low morphological probability and in red for high morphological probability. Three pairs with extreme values of cross-dialectal similarity mismatching between sets were excluded from the analysis (colored in light gray). **(C)** Lower panel: images of unfamiliar shapes.

A list of disyllabic novel ETE pairs was composed using a five-step procedure as described in Appendix 1, based on an SH corpus built by the author (0.2 million words, Wu, in preparation^5^, phonology according to Duanmu, 1999; You, 2013; Zhang and Meng, 2016). The list contained 16 pairs of SH-more-probable and 16 pairs of SH-less-probable ETEs (see Appendix 2). A Standard-Chinese-Shanghainese bi-dialectal male speaker read the list, once in SH and once in SC, yielding 64 recordings, of which silent parts were trimmed. The morpheme frequencies and cross-dialectal similarities are demonstrated in the upper and middle panels of Figure 3.

#### Novel Images

Eight novel shapes were generated with a 24-piece foldable toy (a *Magic Stick*, visual, and conceptual complexities controlled), shot with fixed distance, angle, and illumination, yielding eight different images as shown in the lower panel of Figure 3.

#### Chinese Written Forms (Learning Phase Only)

In the learning phase, in addition to the aurally given novel names and associated images, ideographic Chinese characters for the novel names were also given. They were included because both SH and SC have too many homophonic morphemes (but written with different characters) and the experimental manipulation requires the specific morphemes to be activated during the learning phase.

### Pre-processing of Naming Data

A sound-pressure-based Praat script (Praat software ©, Boersma, 2002; a *post-hoc* voice key, adapted from Pacilly, 2010) was used to automatically detect the start and the end of speech in each recording, which was later screened and corrected. *Response* (whether or not a participant gave a response for a given trial) and *accuracy* were also marked.

## Results

Since the (subjective) *cross-dialectal similarity* data were used in the modeling of identification and naming data, they were first analyzed in Cross-Dialectal Similarity sections. Accuracies and Reaction Times in the Identification Task and Response Rates, Accuracies, and Reaction Times in the Naming Task sections examined responses in the identification and naming task, respectively.

### Cross-Dialectal Similarity

First, to test the influence of *dialectal background* on cross-dialectal similarity rating data, Gaussian Linear Mixed Effect (LME) models (Bates et al., 2013; R Core Team, 2019) were built on raw and by-participant-normalized data. *Participant* and *word* as random intercepts were both highly significant, $\chi_{raw\sim 1|participant}^{2}$ = 3149.5, *p*_1|*participant*_ < 0.001, $\chi_{raw\sim 1|word}^{2}$ = 721.95, *p*_1|*word*_ < 0.001, $\chi_{norm\sim |participant}^{2}$ = 280.74, *p*_*norm*~1|*participant*_ < 0.001, $\chi_{1|word}^{2}$ = 770.33, *p*_1|*word*_ < 0.001. Neither group of bi-dialectals differed significantly from the monolectals in their overall effect of cross-dialectal rating, whether with raw ratings, *t*_*raw*~*SC*−*SHbi*−*dialectal*_ (0.22) = 0.53, *p* = 0.6, *t*_*raw*~*SC*−*ODbi*−*dialectal*_ (0.22) = 0.91, *p* = 0.38, or by-participant normalized ratings, *t*_*norm*~*SC*−*SHbi*−*dialectal*_ (0.05) = 1.06, *p* = 0.29, *t*_*norm*~*SC*−*ODbi*−*dialectal*_ (0.05) = 0.82, *p* = 0.41. *Post-hoc* Least Square Contrasts from *lmerTest* package (Kuznetsova et al., 2013) also confirmed the absence of group-dependent overall difference on similarity ratings.

**Figure 4:**
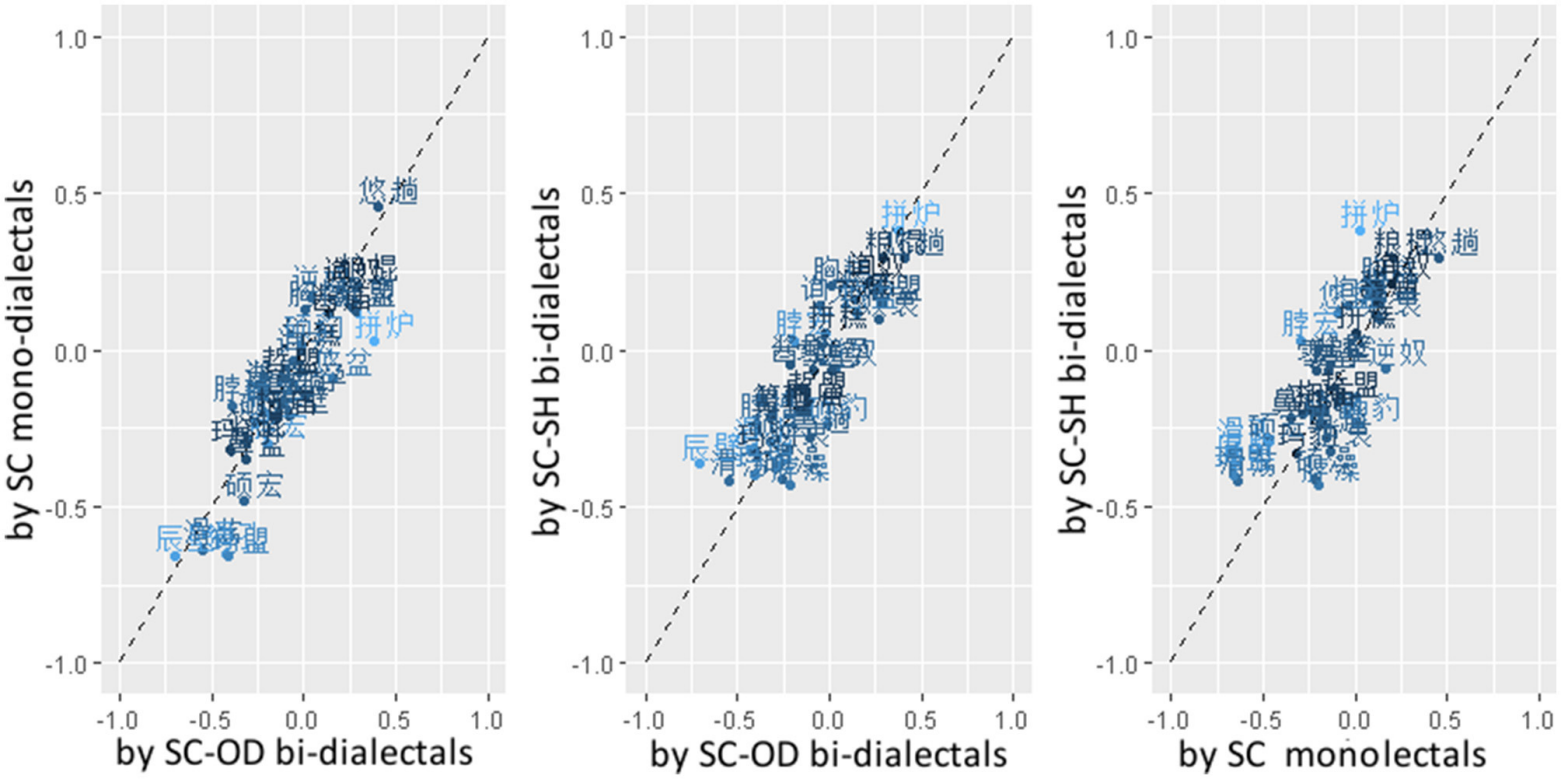
Scatter plots for scaled cross-dialectal similarity ratings of novel ETE pairs (represented with Chinese characters), by SC mono-dialectals (along the vertical axis in the left panel and the horizontal axis in the right panel), SC-SH bi-dialectals (along the vertical axes in the middle and right panels), SC-OD bi-dialectals (along the horizontal axes in the left and middle panels). The deeper color indicates higher consistency.

Second, based on the by-participant-normalized rating data, separate values of *cross-dialectal similarity* were calculated for each pair of novel ETEs, as shown in Figure 4. The average cross-dialectal similarity data for each pair of ETEs showed strong by-pair *Pearson* correlations across all three participant groups, *R*_*SCmonolectal*~*SC*−*SHbi*−*dialectal*_ = *0.78, R*_*SCmonolectal*~*SC*−*ODbi*−*dialectal*_ = *0.89, R*_*SC*−*SHbi*−*dialectal*~*SC*−*ODbi*−*dialectal*_ = *0.87*, indicating high consistency across groups. Figure 4 also shows standard deviations of ratings calculated across groups for each ETE pair with color depth, which indicate the consistency of ratings across groups.

Considering the high consistency across groups, scaled mean cross-dialectal similarity was calculated for each pair of SC-SH novel ETEs and used as a predictor in the following analysis. Three pairs of ETEs (as marked with light gray in the middle panel of Figure 3) were excluded from the analyses to make the distribution of cross-dialectal similarity more comparable between the SH-more-probable and SH-less-probable sets.

### Accuracies and Reaction Times in the Identification Task

We analyzed accuracies (whether each aurally presented stimulus was correctly identified with its corresponding image) and log RTs (reaction times which were natural-log-transformed to improve the distribution) of the correct responses from the identification task with logistic and Gaussian LME models, respectively (Bates et al., 2013). The models included *scaled mean cross-dialectal similarity for each pair of SC-SH novel words* (hereafter referred to as *cross-dialectal similarity*), *the morphological probability of the novel word in SH* (more/less probable, also corresponding to more/less concrete meanings of the morphemes), a *dialect of operation* (SC = source dialect, SC/SH = recipient dialect, SH), the exterior *context provided by the distractor* (high/low-competition for the word trials and inference possible/impossible for the noise trials), and *dialectal background of the participant* (SC monolectal/SC-SH bi-dialectal/SC-OD bi-dialectal) as fixed predictors and included them in all the candidate-models. Besides these crucial predictors, their possible interactions, as well as sociolinguistic predictors, i.e., the *scaled age* and *scaled subjective proficiency* of the participants *in SC*, and their multi-way interactions were also included as candidates of fixed predictors. *By-participant* random intercepts (nested under dialectal background or not), *by-word* random intercepts (nested under morphological probability in SH or not), *by-target-image* random intercepts, and *by-distractor-image* random intercepts were included as the candidates for the random terms. Since the stimuli were counterbalanced across participants to control the repetition of the same novel words, no random slope was possible in the modeling. The structure of the terms in the models reported here was selected *via* model comparison based on Akaike's Criteria (Sakamoto and Ishiguro, 1986). In the process of model comparison, the main effects of the crucial predictors were always included, while the other fixed and random terms were allowed to be excluded according to the result of the model comparison. The model estimates were calculated with the *lsmeans* function from the *lmerTest* package (Kuznetsova et al., 2013).

Responses to words and noises were analyzed separately, as the corresponding mental mechanisms were very different. We here first present results for words.

#### Responses to Word Trials in the Identification Task

**Figure 5:**
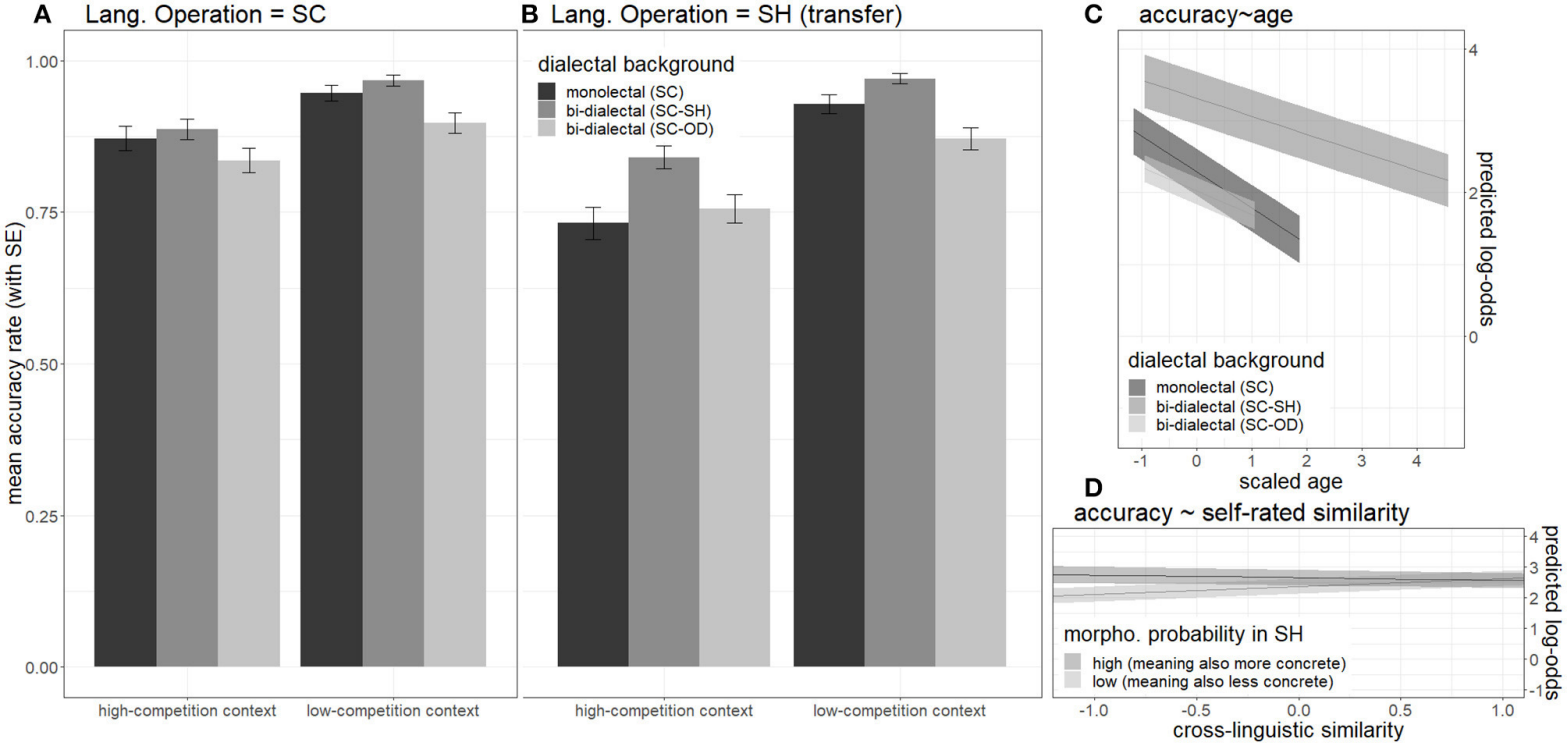
Effects on mean accuracy rates in response to word trials during lexical identification test, including (1) effects of *dialectal background* (color) and *context provided by the distractor* (cluster), in SC **(A)** (left panel) and SH **(B)** (middle panel), (2) the effect of age **(C)** (upper right panel), and (3) the interaction between subjective *cross-dialectal similarity* and *semantic concreteness* **(D)** (lower right panel).

(1) For the accuracy model (logistic LME), the selected random predictors were *by-participant* and *by-target-image* random intercepts, χ*2*_1|*participant*_ = 144.94, *p*_1|*participant*_ < 0.001, χ*2*_1|*target*−*image*_ = 19.86, *p*_1|*target*−*image*_ < 0.001. The crucial terms were all kept according to the design. *SH as dialect of operation, z*_*SH*(*borrowing*)_ = −3.71, *p* < 0.001, *older age, z*_*SH*(*olderage*)_ = −2.63, *p* < 0.05, and *smaller morphological probability in SH (less concrete meanings), z*_*lessprob*.(*lessconcrete*)_ = −2.17, *p* < 0.05, have significant negative main effects on accuracies. *SC-SH dialectal background, z*_*SC*−*SHbi*−*dialectal*_ = 2.70, *p* < 0.05, and *low-competition context, z*_*low*−*competition*_ = 10.98, *p* < 0.001, have positive main effects on accuracies. Main effects of the other predictors were insignificant. The only significant interaction was between smaller *morphological probability in SH* (less concrete meanings) and *cross-dialectal similarity, z*_*lessprob*.(*lessconcrete*):*similarity*_ = 2.25, *p* < 0.05. All the conditions yielded accuracy rates above 70%, as shown in the left panel of Figure 5.

Since some of the SC-SH bi-dialectals were older, separate logistic-LME models were built again for participants with different dialectal backgrounds to further investigate the age effect. Although the effect of age was consistently negative for all three groups, it only reached significance for the SC monolectals, *z*_*SCmonolectals*:*age*_ = −2.51, *p* < 0.05, and did not reach significance for the SC-SH bi-dialectals, *z*_*SC*−*SHbi*−*dialectals*:*age*_ = −1.87, *p* =0.06 (marginally significant), or the SC-OD bi-dialectals, *z*_*SC*−*ODbi*−*dialectals*:*age*_ = −0.77, *p* =0.06. Also, after excluding the data from the SC-SH bi-dialectals who were older than the oldest of the other two groups, the age effect within this group turned totally insignificant, *z*_*SC*−*SHbi*−*dialectals*(*young*):*age*_ = −0.3, *p* =0.77. Thus, bi-dialectals were less affected by age than monolectals.

**Figure 6:**
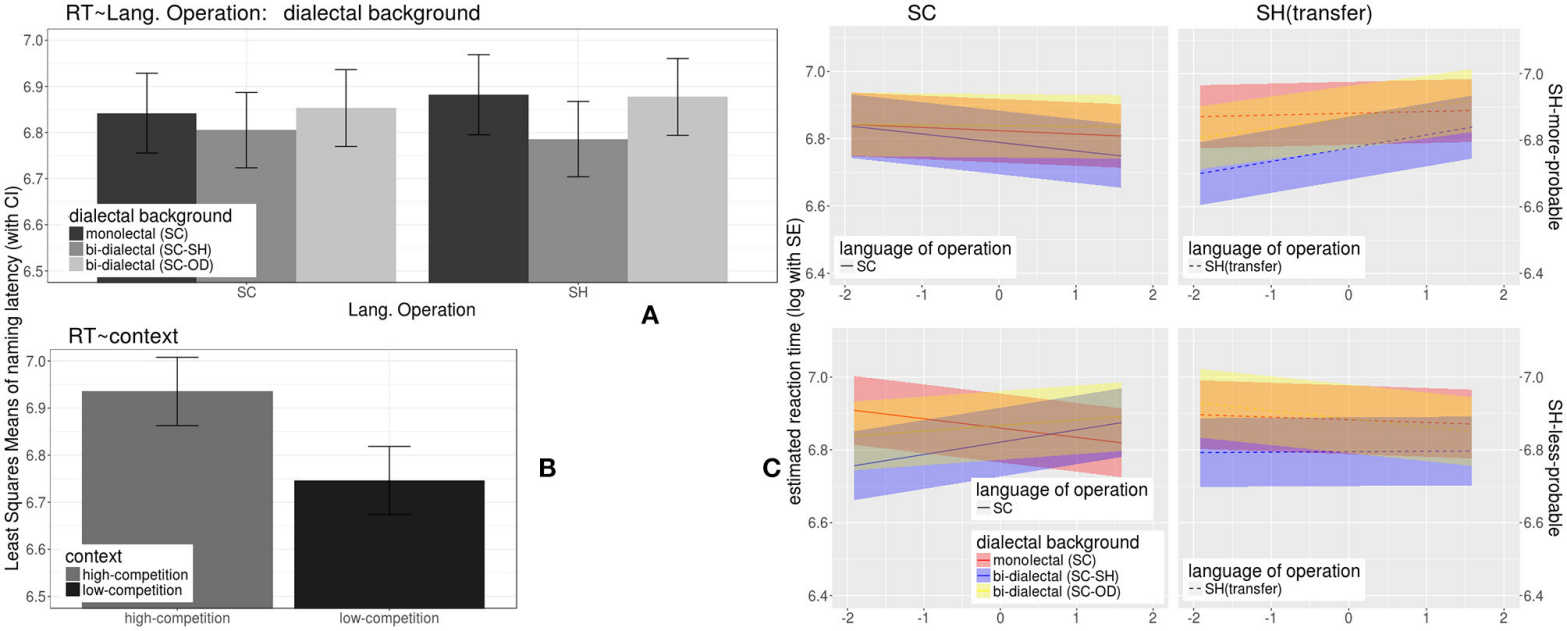
Effects on reaction times in (correct) response to word trials during lexical identification test, modeled with LME method, including (1) the effects of *dialectal background* (color), in SC **(A)** (upper left panel, left cluster) and SH **(A)** (upper left panel, right cluster), (2) the effect of *context provided by the distractor* **(B)** (lower left panel), and (3) the linear interaction **(C)** (the four-figure grid in the right panel) of *dialectal background* (color), *cross-dialectal similarity* (along the vertical axes), *the novel word's morphological probability in SH* (upper vs. lower subplots), and *language of operation* (left vs. right subplots).

(2) For the RT data of the correctly identified trials, statistics of LME models are shown in Appendix 3 with Satterthwaite approximation (Kuznetsova et al., 2013). The model estimates (lsmeans function, lmerTest package, Kuznetsova et al., 2013) are demonstrated in Figure 6.

As shown in the right panel of Figure 6, the three-way interaction across the *dialectal background, morphological probability in SH (also reflecting concreteness)*, and *cross-dialectal similarity* were complicated and subtle, with large variation along the similarity dimension. Thus, Generalized Additive Models (GAMs) were built using the “mgcv” package (Wood, 2006, 2011) in R (R Core Team, 2019) to investigate the potential nonlinear interactions between these predictors. The candidate models included *log RTs* as the dependent variable, and linear and smooth predictors were included, as described in more detail in the following.

**Figure 7:**
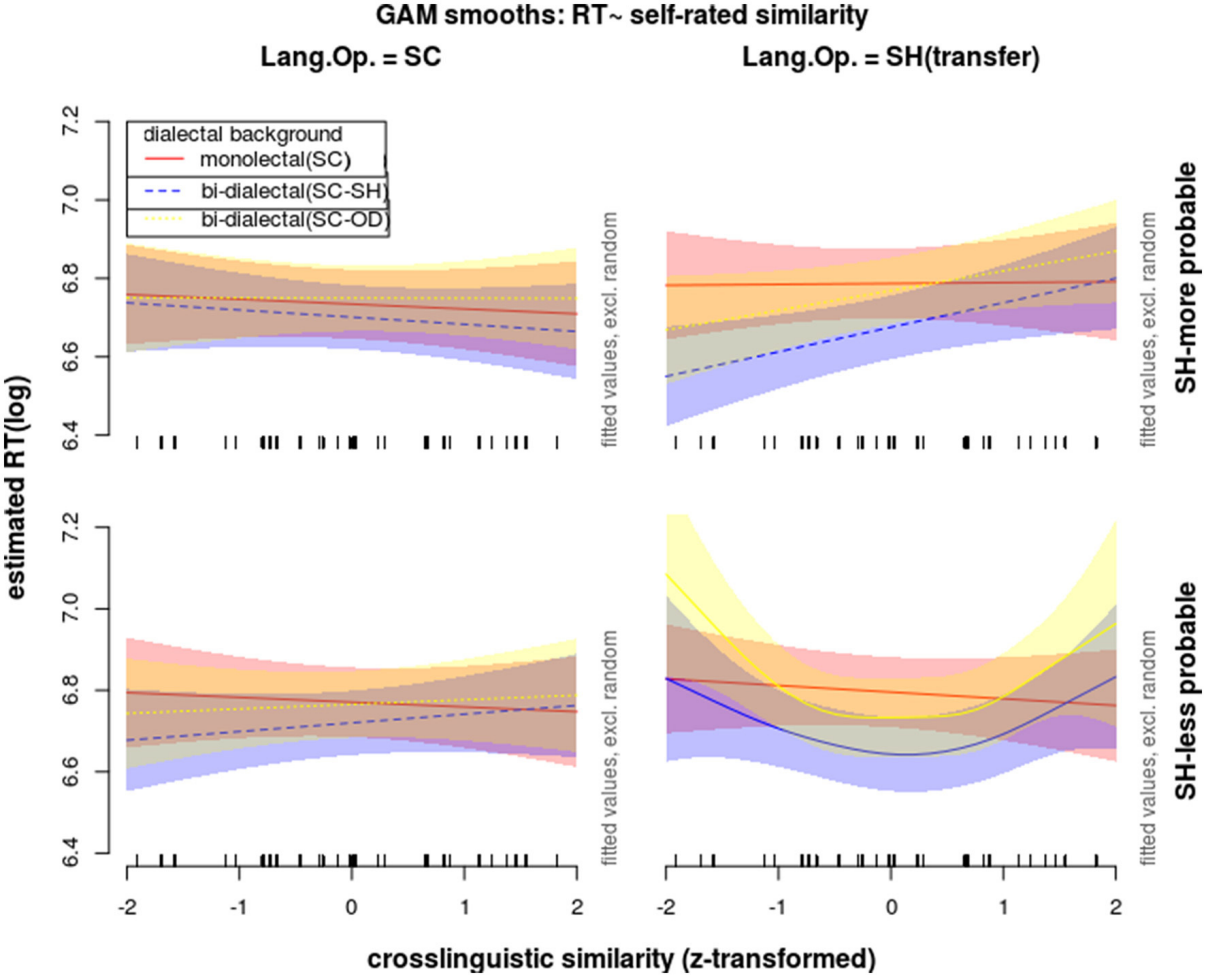
Effects on reaction times in correct response to word trials during the lexical identification test, modeled with GAM, showing non-linear interactions of *dialectal background* (color), *cross-dialectal similarity* (along the vertical axes), *the novel word's morphological probability in SH* (upper = high vs. lower = low subplots), and *language of operation* (left = SC vs. right = SH subplots).

Smooth functions were used to model non-linear functional relations between RTs and *cross-dialectal similarity*. The factorial predictors, namely *dialectal background, dialect of operation*, and *the novel word's morphological probability in SH (also reflecting concreteness)* were combined into a twelve-level predictor (SC monolectal/SC-SH bi-dialectal/SC-OD bi-dialectal × SC/SH × more/less probable) and this new predictor was included in both the fixed linear predictors and fixed smoothes. Besides this crucial predictor, the *context provided by the distractor* was also included as a fixed linear predictor. The candidates for random predictors were *participant, word, target-image*, and *distractor-image*. The structure of the final model was decided by model comparison based on the Akaike Information Criterion likelihood values (Sakamoto and Ishiguro, 1986). After the structure of the model was decided, auto-correlation values were calculated based on the trial order of data points, which turned out to be close to 0. Thus, there was no need to build an AR1 error model (Wood, 2006, 2011). The coefficients for the parametric predictors and the *F*-statistics for the smooth terms are shown in Table 3. The results are depicted in Figures 6, 7.

**Table 3:** GAM model results for recognition data.

Formula: RTadjusted_log ~ s(Similarity, by = LanBGLanOpMfreq_Tst, k = 18) +LanBGLanOpMfreq_Tst + Context + s(Similarity, Participant, bs = “fs”, m = 1) + s(Word, bs = “re”) + s(TargetImg, bs = “re”) (Signif. codes: 0 ‘***' 0.001 ‘**' 0.01 ‘*' 0.05 ‘.' 0.1 ‘' 1, R-sq.(adj) = 0.42, Deviance explained = 45.8%, -ML = 135.89, Scale est. = 0.055762, n = 3131)					
- LanBGLanOpMfreq_Tst: a combined predictor of ***dialectal background**, **dialect of operation***, and ***the novel word's morphological probability in SH (also reflecting concreteness)***					
- Similarity: cross-dialectal similarity					
- Context: ***context provided by the distractor***					
- Participant: ***participant***					
- Word: ***word***					
- TargetImg: ***target-image***					
	**Estimate**	**Std. error**	***t*** **value**	**Pr(>|t|)**	
**Parametric coefficients**					
(Intercept)	6.94	0.04	161.94	<2e−16	***
LanBGLanOpMfreq_Tst: SC-OD bi-dialectals in SC to SH-less-probable-stimuli	0.02	0.03	0.55	0.58	
LanBGLanOpMfreq_Tst: SC-OD bi-dialectals in SH to SH-more-probable-stimuli	0.02	0.03	0.67	0.50	
LanBGLanOpMfreq_Tst: SC-OD bi-dialectals in SH to SH-less-probable-stimuli	0.05	0.03	1.82	0.07	.
LanBGLanOpMfreq_Tst: SC monolectals in SC to SH-more-probable-stimuli	– 0.02	0.05	– 0.32	0.75	
LanBGLanOpMfreq_Tst: SC monolectals in SC to SH-less-probable-stimuli	0.02	0.05	0.44	0.66	
LanBGLanOpMfreq_Tst: SC monolectals in SH to SH-more-probable-stimuli	0.04	0.05	0.77	0.44	
LanBGLanOpMfreq_Tst: SC monolectals in SH to SH-less-probable-stimuli	0.05	0.05	0.94	0.35	
LanBGLanOpMfreq_Tst: SC-SH bi-dialectals in SC to SH-more-probable-stimuli	– 0.05	0.04	– 1.08	0.28	
LanBGLanOpMfreq_Tst: SC-SH bi-dialectals in SC to SH-less-probable-stimuli	– 0.03	0.05	– 0.65	0.52	
LanBGLanOpMfreq_Tst: SC-SH bi-dialectals in SH to SH-more-probable-stimuli	– 0.07	0.05	– 1.65	0.10	.
LanBGLanOpMfreq_Tst: SC-SH bi-dialectals in SH to SH-less-probable-stimuli	– 0.05	0.05	– 1.16	0.25	
Context: low-competition	– 0.19	0.01	– 22.40	<2e– 16	***
	**edf**	**Ref.df**	***F***	***p*** **value**	
**Approximate significance of smooth terms**					
s(Similarity) by LanBGLanOpMfreq_Tst: SC-OD bi-dialectals in SC to SH-more-probable-stimuli	1.00	1.00	0.00	0.99	
s(Similarity) by LanBGLanOpMfreq_Tst: SC-OD bi-dialectals in SC to SH-less-probable-stimuli	1.00	1.00	0.16	0.69	
s(Similarity) by LanBGLanOpMfreq_Tst: SC-OD bi-dialectals in SH to SH-more-probable-stimuli	1.00	1.00	3.56	0.06	.
s(Similarity) by LanBGLanOpMfreq_Tst: SC-OD bi-dialectals in SH to SH-less-probable-stimuli	2.75	3.19	3.36	0.02	*
s(Similarity) by LanBGLanOpMfreq_Tst: SC monolectals in SC to SH-more-probable-stimuli	1.00	1.00	0.25	0.62	
s(Similarity) by LanBGLanOpMfreq_Tst: SC monolectals in SC to SH-less-probable-stimuli	1.00	1.00	0.19	0.66	
s(Similarity) by LanBGLanOpMfreq_Tst: SC monolectals in SH to SH-more-probable-stimuli	1.00	1.00	0.01	0.94	
s(Similarity) by LanBGLanOpMfreq_Tst: SC monolectals in SH to SH-less-probable-stimuli	1.00	1.00	0.37	0.54	
s(Similarity) by LanBGLanOpMfreq_Tst: SC-SH bi-dialectals in SC to SH-more-probable-stimuli	1.00	1.00	0.60	0.44	
s(Similarity) by LanBGLanOpMfreq_Tst: SC-SH bi-dialectals in SC to SH-less-probable-stimuli	1.00	1.00	0.72	0.40	
s(Similarity) by LanBGLanOpMfreq_Tst: SC-SH bi-dialectals in SH to SH-more-probable-stimuli	1.00	1.00	6.11	0.01	*
s(Similarity) by LanBGLanOpMfreq_Tst: SC-SH bi-dialectals in SH to SH-less-probable-stimuli	2.17	2.52	2.37	0.06	.
s(Similarity, Participant)	161.42	334.00	5.62	<2e−16	***
s(Word)	9.85	25.00	1.02	0.00	***
s(TargetImg)	6.50	7.00	25.09	<2e−16	***

None of the parametric coefficients in Table 3 was significant. Only SC-SH bi-dialectals' RTs to more probable SH trials were marginally shorter than average, *t* = −1.65, *p* =0.1, and SC-OD bi-dialectals' RTs to less probable SH trials were marginally longer than average, *t* = 1.82, *p* =0.07.

However, when the *dialect of operation* was SH (i.e., under the condition of borrowing), as shown by the colored curves and bands (Van Rij et al., 2015) in Figure 7 and by the F-statistics for the smooth terms in Table 3, while monolectals showed no sensitivity to cross-dialectal similarity in all the correct trials, the two groups of bi-dialectals' RTs in correct SH trials were influenced by cross-dialectal similarity in a non-linear way, *F*_*SC*−*SHbi*−*dialectal*:*more*−*prob*._(1) = 6.11, *p* < 0.05, *F*_*SC*−*SHbi*−*dialectal*:*less*−*prob*._(2.52) = 2.37, *p* =0.06, *F*_*SC*−*ODbi*−*dialectal*:*more*−*prob*._(1) = 3.561, *p* =0.06, *F*_*SC*−*ODbi*−*dialectal*:*less*−*prob*._(3.19) = 3.36, *p* < 0.05.

**Figure 8:**
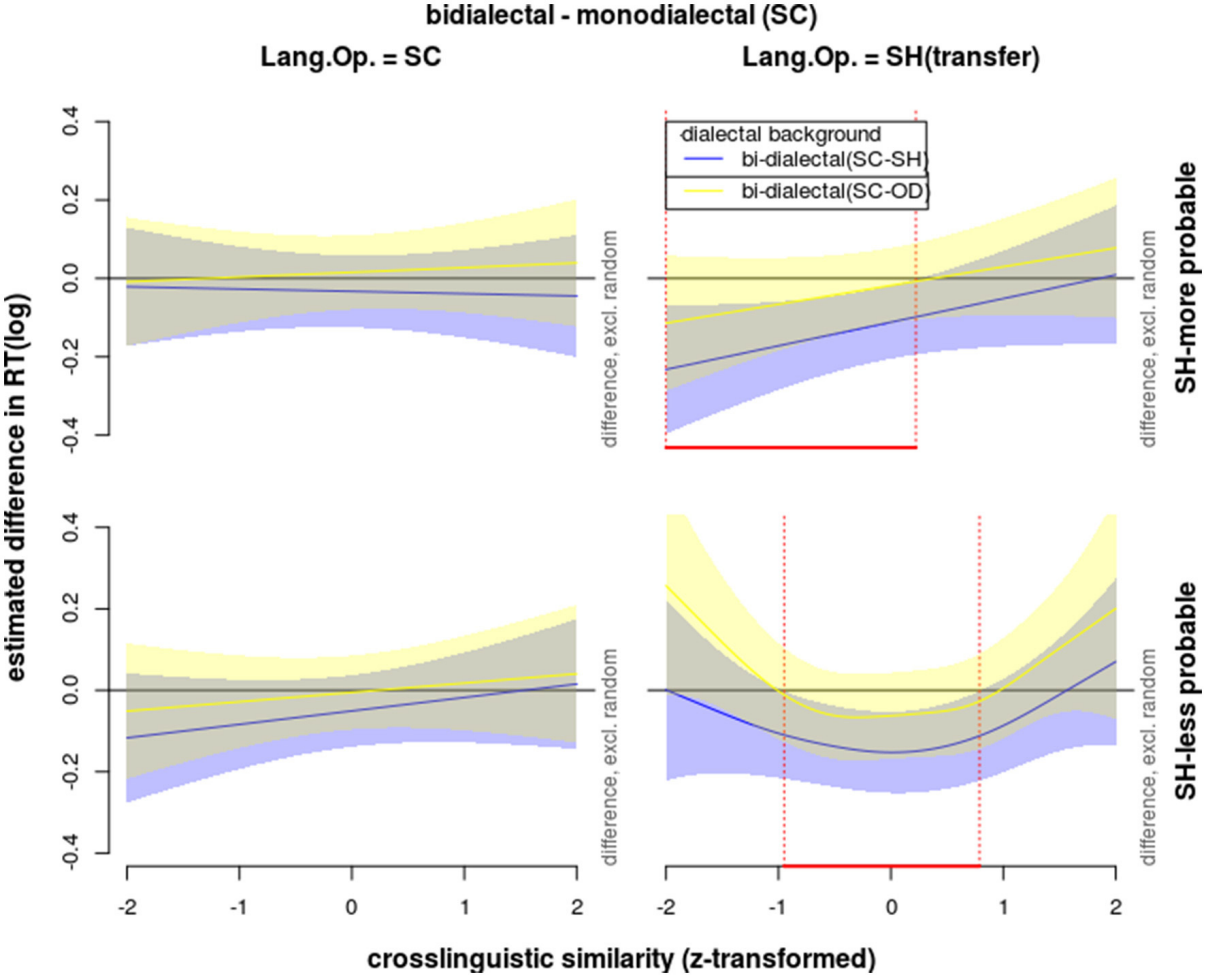
Effects on reaction times in correct responses to word trials during the lexical identification test, modeled with GAM, showing the estimated difference curves comparing SC-SH (blue) and SC-OD bi-dialectals (yellow) against SC mono-dialectals, taking into consideration interactions of *cross-dialectal similarity* (along the vertical axes), *the novel word's morphological probability in SH* (upper = high vs. lower = low subplots), and *language of operation* (left = SC vs. right = SH subplots).

Since the F-statistics for the smooth terms compares each manipulation level with the average level, the two bi-dialectal groups were further compared with the monolectal group *post-hoc*. The estimated differences were depicted in Figure 8 using the *plot_diff* function from *itsadug* R package (Van Rij et al., 2015). The parts of the curves showing significant differences between bi-dialectals and monolectals were marked with vertical dash lines.

In response to morphologically more probable SH stimuli, SC-SH bi-dialectals' RTs increased with cross-dialectal similarity, while their responses to morphologically less probable SH stimuli showed a concave shape of nonlinear correlation with cross-dialectal similarity. SC-OD bi-dialectals also showed non-linear patterns (yellow lines and bands in Figures 6, 7), which did not reach significance but are shape-wise more in line with SC-SH bi-dialectals than with SC monolectals.

Taking together the results reported in Responses to Word Trials in the Identification Task section and Figures 4–7, in the identification test, some effects took place by all three groups of participants. (1) All three groups were able to identify the referent of loan forms in SH with considerable accuracy. (2) Age showed a negative effect on accuracies but did not affect RTs of correct responses. (3) All three groups responded more accurately to more concrete words. (4) All three groups gave less accurate responses to the SH loan forms than to the original SC forms. (5) Low-competition context increased accuracies and reduced RTs.

The three groups of participants showed significant differences in SH, namely under the condition of cross-dialectal borrowing. (6) Only non-SH participants took longer to correctly identify SH forms (novel loanwords) than SC form (source forms). In contrast, SC-SH bi-dialectals were even slightly faster when responding to SH forms. (7) SC monolectals showed no sensitivity to *cross-dialectal similarity* regardless of a *dialect of operation*. In contrast, the bi-dialectals were not sensitive to a *cross-dialectal similarity* in SC but turned sensitive when the *dialect of operation* switched to SH (comprehension borrowing). Also, only the bi-dialectals showed non-linear interactions between cross-dialectal similarity and morphological probability (lower right panel of Figure 8).

#### Responses to Noise Trials in the Identification Task

Regarding the noise trials, for the *accuracy* model, the selected random predictors were by-participant and by-distractor-image random intercepts, $\chi_{1|participant}^{2}$ = 89.49, *p*_1|*participant*_ <0.001, $\chi_{1|distractor-image}^{2}$ = 4.81, *p*_1|*distractor*−*image*_ <0.001. *SC-OD bi-dialectal background, z*_*SC*−*ODbi*−*dialectal*_ = −3.29, *p* < 0.001, *older age, z*_*age*_ = −3.72, *p* < 0.001, and *inference-impossible context*, z _inferenceimpossible_ = −6.03, *p* < 0.001, showed significant negative main effects on accuracies. Main effects of the other predictors were insignificant. Inference-impossible context had a significant negative interaction with SC-OD as *dialectal background, z*_*inferenceimpossible*:_ _*SC*−*ODbi*−*dialectal*_ = −3.53, *p* < 0.001, as well as a significant positive interaction with *age, z*_*inferenceimpossible*:*age*_ = 4.67, *p* < 0.001. The three-way interaction across inference-impossible context, SC-OD as *dialectal background*, and *age* was also significant, *z*_*inferenceimpossible*:_ _*SC*−*ODbi*−*dialectal*:*age*__=_ −2.19, *p* < 0.05.

**Figure 9:**
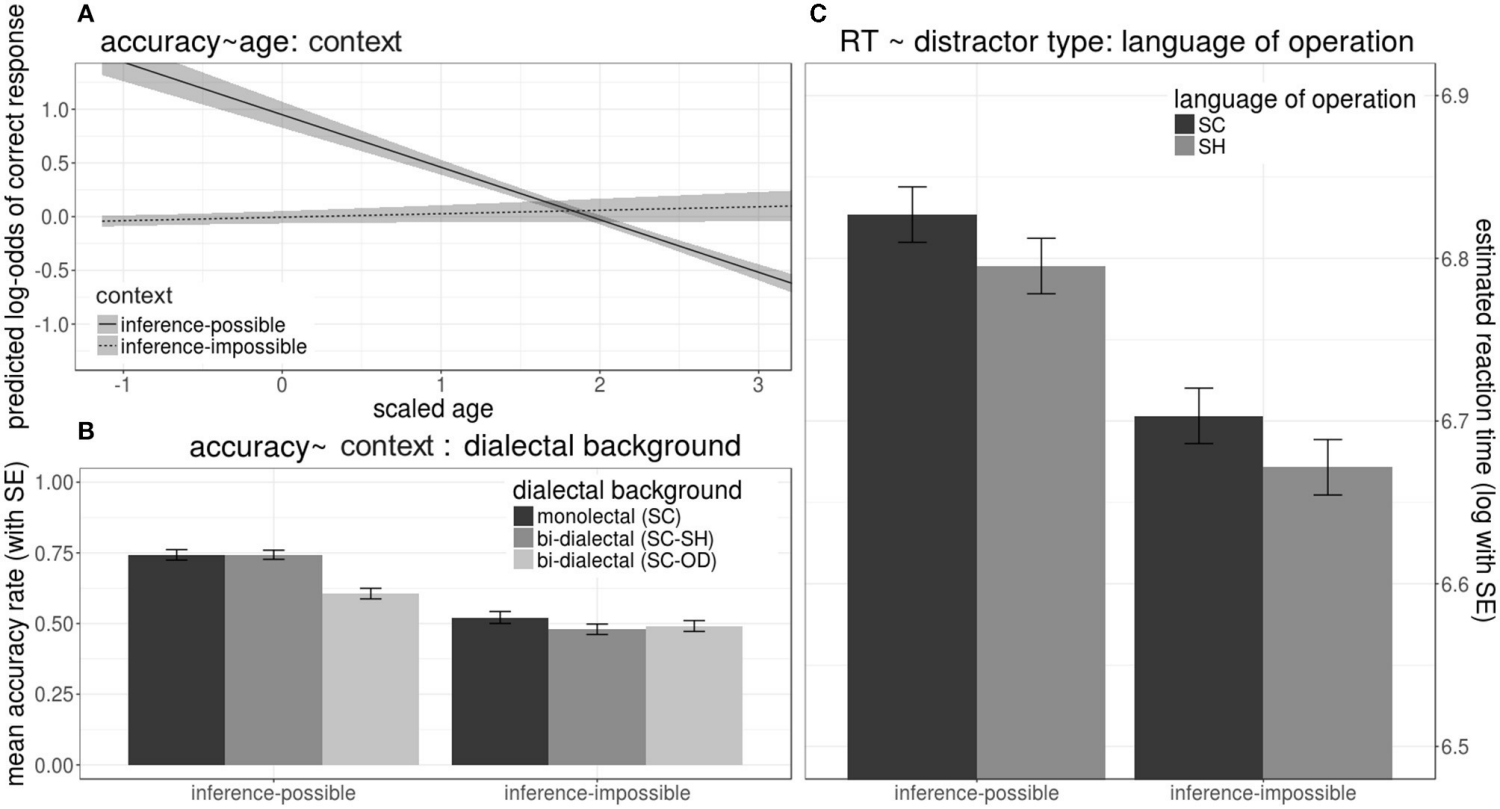
Effects on mean accuracy rates **(A,B)** (left panels) and reaction times **(C)** (right panels) in response to noise trials during lexical identification test, including (1) interaction of *age* and *context provided by the distractor* on accuracies (upper left panel), (2) interaction of *dialectal background* and *context provided by the distractor* on accuracies (lower left panel), (3) effects of *dialectal background* and *context provided by the distractor* on reaction times (right panel).

As shown in the lower-left panel of Figure 9, given an inference-possible context, all groups' mean accuracies were above 60%, which in contrast dropped to chance level (about 50%) when the context was inference-impossible. Accuracies in noise trials dropped with the increase of age, but also only when the context was inference-possible (upper left panel of Figure 9).

For the RT data of correct noise trials, the selected random predictors were by-participant and by-word random intercepts, $\chi_{1|participant}^{2}$ = 1033.30, *p*_1|*participant*_ <0.001, $\chi_{1|\text{word}}^{2}$ = 3.43, *p*_1|word_ =0.06. *SH* as *dialect of operation, t*_*SH*(*borrowing*)_ (2252.41) = −3.03, *p* < 0.001, as well as inference-impossible context, *t*_*inferenceimpossible*_(2245.50)= −11.54, *p* < 0.001, showed significant main effects in reducing RTs. As shown in the right panel of Figure 9, all groups took longer to respond when inference was possible and when *dialect of operation* was SC.

Taken together, most effects in the noise trials were insensitive to the participants' dialectal backgrounds. The only significant effect of *dialectal background* was that SC-OD bi-dialectals were less accurate than SC monolectals and SC-SH bi-dialectals in inference-possible context. However, they showed no difference from the other two groups when inference was impossible.

### Response Rates, Accuracies, and Reaction Times in the Naming Task

As noted in Holistic Casting versus Morpheme-Based Re-Encoding in Lexical Borrowing section, the experiment compared four conditions of prior short-term exposure conditions (*learning experience*), where the participants had either *bi-dialectal* or *monolectal exposure* to the target words prior to the naming and were tested either in *SC* or *SH*. Hence, besides the crucial predictors which were also tested with the identification data, one additional predictor, ***learning experience***, was included in models for the naming data. All the other settings were identical to Accuracies and Reaction Times in the Identification Task section.

#### Response Rates in the Naming Task

Regarding whether or not participants gave responses in the naming task, the selected random predictors were by-participant and by-target-image random intercepts, $\chi_{1|participant}^{2}$ = 66.51, *p*_1|*participant*_ <0.001, $\chi_{1|target-image}^{2}$ = 60.95, *p*_1|*target*−*image*_ <0.001.With incidental *bi-dialectal exposure and tested in SC* as the base-line (i.e., compared with the richest lexical experience plus the easiest testing condition), monolectal learning experience significantly reduced response rates, whether the test dialect was SC or SH (indirect exposure), *z*_*exposureonlyinSC&testedinSC*_ = −2.60, *p* < 0.05, *z*_*exposureonlyinSC&testedinSH*_ = −5.67, *p* < 0.001. Regarding *dialectal background*, SC-SH bi-dialectals were more likely to give responses than SC monolectals, z _*SC*−*SHbi*−*dialectal*_= 3.87, *p* < 0.001; however, they were less likely to give responses to SH less-probable words than to SH more-probable words, z _*SC*−*SHbi*−*dialectal*:*SHless*−*prob*._ = −2.07, *p* < 0.05.

**Figure 10:**
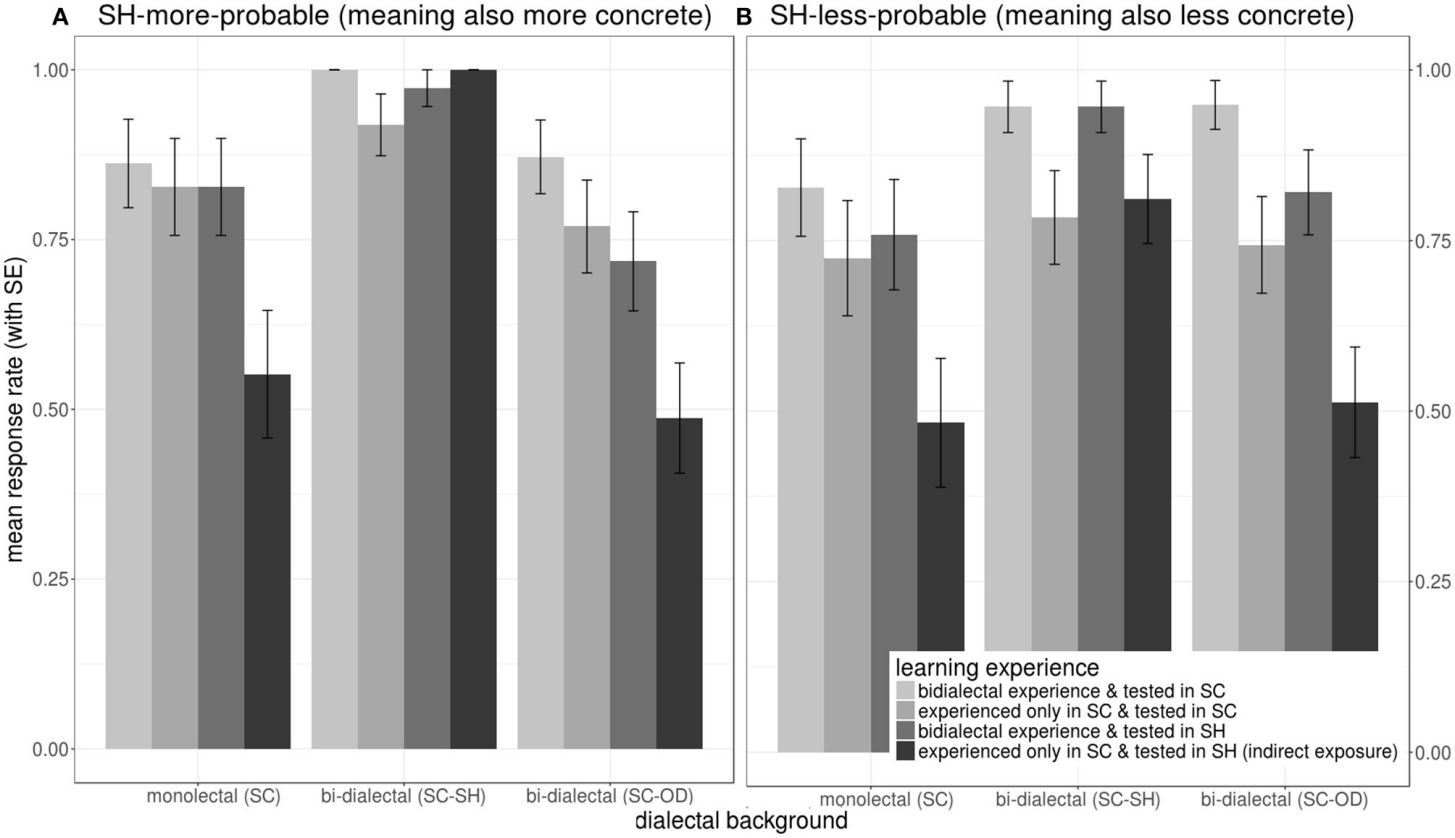
Effects on mean response rates during the lexical production test, showing interactions of the *learning experience* (a combination of the participants' exposure to target words prior to the naming task and *dialect of operation in the naming task*), (color) and *dialectal background* (cluster), in response to SH-more-probable **(A)** (left panel) and SH-less-probable **(B)** (right panel) stimuli.

As shown in Figure 10, except for the *indirect exposure* condition, mean response rates were all above 0.5 across conditions. Note that SC-SH bi-dialectals were the only group whose responses rates were significantly influenced by morphological probabilities.

#### Accuracies in the Naming Task

Regarding accuracy during naming, the selected random predictors were by-participant, by-word, and by-target-image random intercepts, $\chi_{1|participant}^{2}$ = 83.84, *p*_1|*participant*_ <0.001, $\chi_{1|word}^{2}$ = 4.10, *p*_1|*word*_ <0.05, $\chi_{1|target-image}^{2}$ = 60.20, *p*_1|*target*−*image*_ <0.001. SH-less-probable and also less-concrete targets elicited less accurate responses across all three groups, z _*less*−*prob*.(*lessconcrete*)_ = −2.80, *p* < 0.01, hence this is mainly an effect of concreteness. With *bi-dialectal exposure and tested in SC* as the base-line, SH as dialect of operation significantly reduced accuracy, *z*_*bi*−*dialectalexposure&testedinSH*_ = −2.12, *p* < 0.05, z _*exposureonlyinSC&testedinSH*(*indirectexposure*)_ = −6.27, *p* < 0.001. However, SC-SH bi-dialectal background showed a significant positive interaction with indirect exposure, *z*_*SC*−*SHbi*−*dialectal*:*exposureonlyinSC&testedinSH*(*indirectexposure*)_ = 3.13, *p* < 0.001.

**Figure 11:**
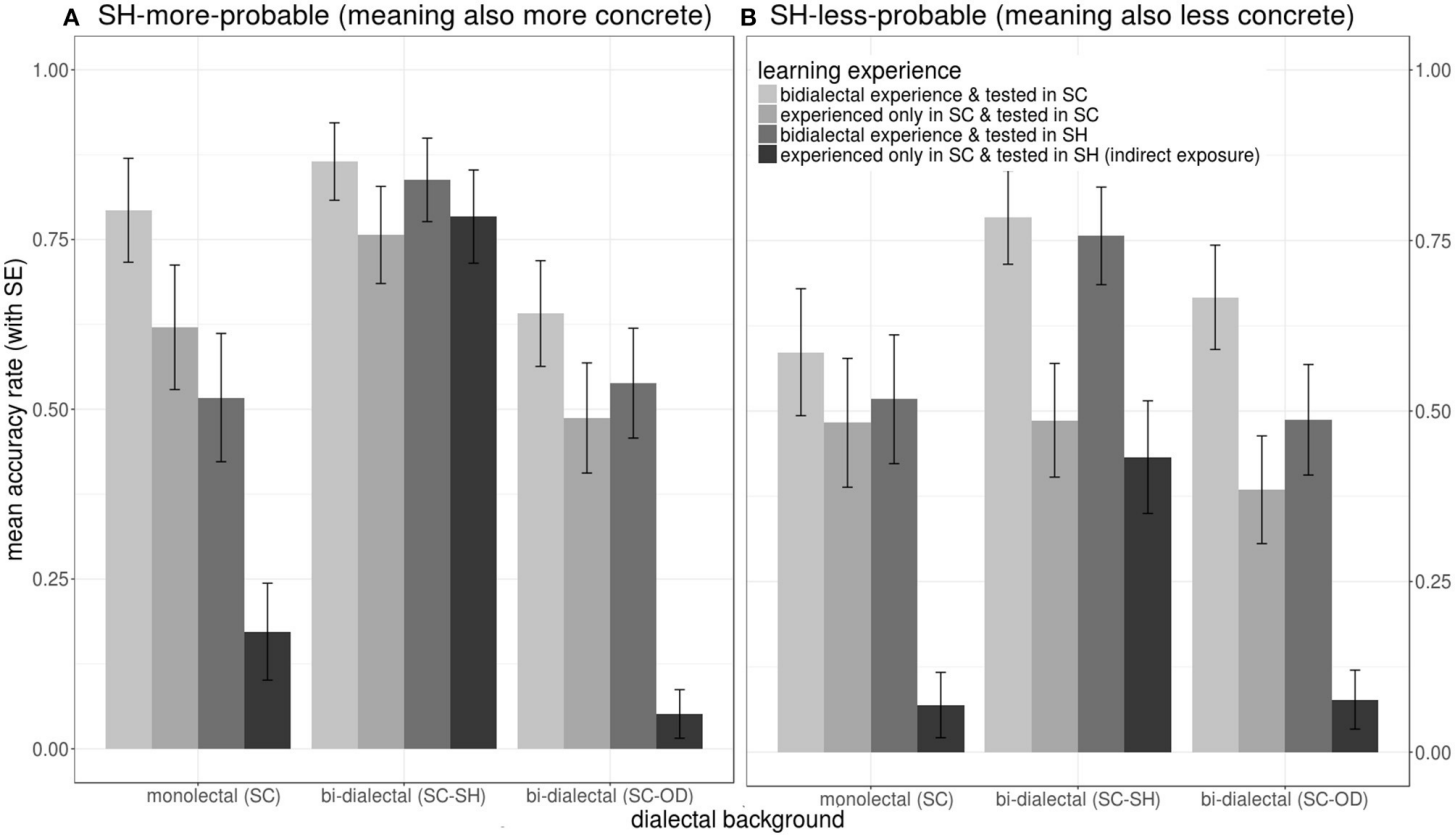
Effects on mean accuracies during the lexical naming test, showing interactions of the *learning experience* (a combination of the participants' *exposure to target words* prior to the naming task and *language of operation in the naming task*, color) and *dialectal background* (cluster), in response to SH-more-probable **(A)** (left panel) and SH-less-probable **(B)** (right panel) stimuli.

As shown in Figure 11, long-term SH-specific background is critical for production accuracy. (1) *SH* as *dialect of operation* significantly reduced *non-SH* participants' but not *SC-SH bi-dialectals'* naming accuracy. (2) While bi-dialectal exposure significantly increased accuracy across all participant groups, unlike the non-SH groups, SC-SH bi-dialectals suffer little from the lack of prior direct exposure to the target SH forms, especially when SH morphological probability was high. (3) Regarding non-SH groups, lower *concreteness* reduced their mean accuracies only in SC but not in SH. In contrast, SC-SH bi-dialectals named *SH-more-probable* words more accurately than *SH-less-probable* words in both dialects.

#### Reaction Times in the Naming Task (i.e., Naming Latencies)

Regarding RTs in correct naming, the selected random predictors were by-participant, by-word, and by-target-image random intercepts, χ*2*_1|*participant*_ = 3.60, *p*_1|*participant*_ =0.06, χ*2*_1|*word*_ = 5.63, *p*_1|*word*_ <0.05, χ*2*_1|*target*−*image*_ = 31.16, *p*_1|*target*−*image*_ <0.001. With *bi-dialectal exposure and tested in SC* as the base-line (i.e., compared with the richest experience plus the easiest testing condition), main effects of all three other types of *learning experience* increased naming latencies, *t*_*exposureonlyinSC&testedinSC*_(354.89) = 3.05, *p* < 0.001, t _*exposureonlyinSC&testedinSH*(*indirectexposure*)_(380.26) = 1.73, *p* =0.09, t _*bi*−*dialectalexposure&testedinSH*_(340.85) = 2.83, *p* < 0.001. However, with *SH* as *dialect of operation*, SC-SH bi-dialectals' naming latencies were significantly reduced, *t*_*SC*−*SHbi*−*dialectal*:*bi*−*dialectalexposure&testedinSH*_ (333.32) = −4.16, *p* < 0.001, *t*_*SC*−*SHbi*−*dialectal*:*exposureonlyinSC&testedinSH*(*indirectexposure*)_(370.75) = −2.04, *p* < 0.05, which reversed the interfering main effects. *SC-OD bi-dialectals* also enjoyed a facilitatory effect from bi-dialectal exposure when *dialect of operation* was *SH, t*_*SC*−*ODbi*−*dialectal*:*bi*−*dialectalexposure&testedinSH*_ (340.77) = −2.07, *p* < 0.05.

**Figure 12:**
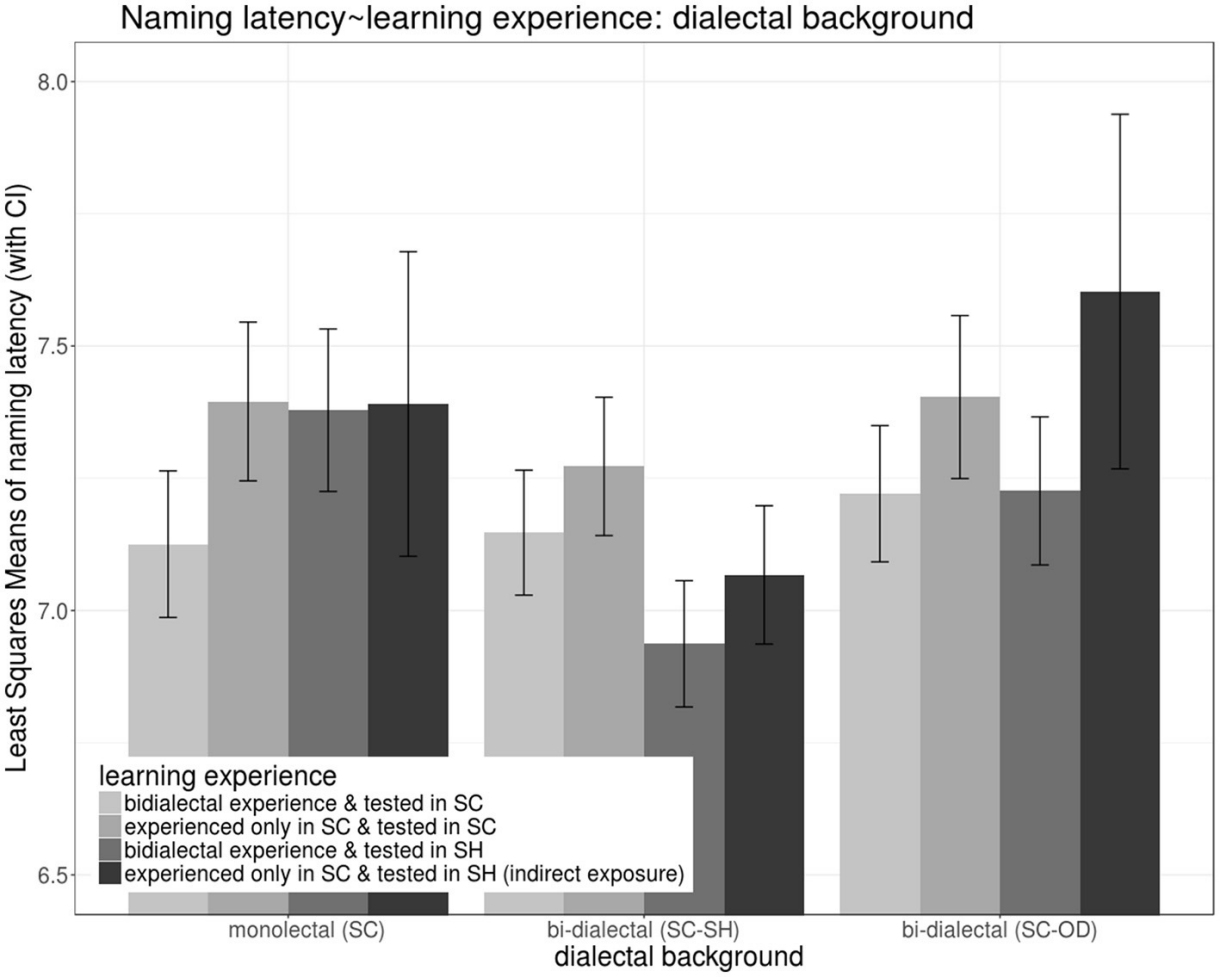
Effects on naming latencies during the lexical production test, showing an interaction between *learning experience* (a combination of the participants' exposure to target words prior to the naming task and *language of operation in the naming task*, color) and *dialectal background* (cluster).

*Post-hoc lsmeans* model estimates (Kuznetsova et al., 2013) in Figure 12 showed the importance of long-term and short-term bi-dialectal experience in naming tasks. (1) Short-term bi-dialectal exposure reduced general SC naming latencies across all groups. However, (2) in the recipient dialect SH, only bi-dialectals were significantly facilitated by bi-dialectal exposure. (3) SC monolectals responded slower in SH than in SC, but SC-SH bi-dialectals responded faster in SH than in SC.

## Discussion

This study investigated the cognitive processes underlying cross-dialectal lexical borrowing in the auditory comprehension and production of bi-dialectals and monolectals. The following five findings shed light on individuals' collective cognitive processes involved in the initial stage of the social emergence of loanwords.

### Long-Term Bi-Dialectism Counteracts Age-Related Reduction of Word-Learning Ability

The comprehension borrowing experiment showed that, whether the stimuli were presented in the source form or the loan form, the accuracy of word identification reduced with age in all three groups of participants, as predicted by the age-related gradual reduction hypothesis (Connor et al., 2006). However, the age-related reduction of bi-dialectals when it comes to lexical-learning ability is smaller and starts at a later age (> 35 years of age). This indicates that long-term bi-dialectism may have protective effects on both word learning and cross-dialectal comprehension borrowing of recently learned novel words.

### Short-Term Bi-Dialectal Exposure Facilitates Production Borrowing by Enriching Instead of Increasing Lexical Exposure

In the production borrowing test, compared with creatively producing nonce loanwords (given source-dialect-only prior exposure), reproducing briefly established loanwords (with short-term bi-dialectal prior exposure) resulted in more responses and higher accuracies. Similarly, short-term bi-dialectal prior exposure also facilitates the production of source word forms. Since the duration of short-term dialectal-general exposure (direct and indirect together) to the target loanwords was held the same in the experiment, this finding suggested that it was enriched instead of increased prior lexical exposure that facilitated production borrowing. The finding is in line with previous research findings that increased lexical variability facilitates word learning (for e.g., see Lively et al., 1993). It also revealed that cross-dialectal comprehension borrowing has beneficial effects in consolidating lexical representations of recently learned novel words in both source and recipient dialects. This reciprocal cognitive benefit can explain the historical linguistic phenomenon that cross-dialectal borrowing seems to be very frequent in bi-dialectal communities with long-term co-evolving dialects (Trudgill, 1986).

### Bi-Dialectals Store New Lexical Items, While Monolectal Assimilate Lexical Variants

As shown by the results of GAM analyses (Figures 6, 7), both groups of bi-dialectals responded more slowly or non-linearly to words with increased cross-dialectal similarity. These effects are in line with previous research findings of cross-linguistic lexical competition and parallel inhibition (for example, see Dijkstra et al., 2010; Wu et al., 2019) and revealed an early existence of lexical-level cross-dialectal competition as loan forms were initially introduced. In contrast, none of such lexical-level effects was found in the monolectals. The facilitatory effects of monolectals of cross-dialectal similarity indicated that they probably performed perceptual assimilation (Best and Strange, 1992) and accent-adaptation (Sumner and Samuel, 2009; Larraza and Best, 2018) mechanisms instead when they encounter cross-dialectal loan forms for the first time.

Note that bi-dialectals without prior recipient-dialect-specific experience showed similar response patterns as bi-dialectals who are speakers of the recipient dialect but differed significantly from source-dialect monolectals. This indicated that the difference in cross-dialectal lexical representation pattern during comprehension borrowing resulted from bi-dialectism in general instead of recipient-dialect-specific experience.

Hence, hearing cross-dialectal loan forms, monolectals tend to temporarily apply perceptual assimilation and accent adaptation mechanisms to process the new “accentual variants” and hence maintain a relatively stable state of the mental lexicon. On the other hand, bi-dialectals readily add new lexical items to their integrated mental lexicon and update their mental lexicons more actively. Zooming out from individual speakers to linguistic communities, one can further imagine that, with co-evolving dialects in context, a larger proportion of bi-dialectals may mean more active and sustainable cross-dialectal lexical borrowing at the community level (see also previously, Poplack et al., 1988 on highly bilingual communities), which may expedite lexical alignment between the two involved dialects (or languages) in linguistic evolution.

### Bi-Dialectals Borrow Compounds Based on Morphemes, While Monolectals Cast Transferred Forms Holistically

Previously, it was found that constituent effects are less likely to surface in production than in comprehension (Janssen et al., 2014). However, the current study found recipient-dialect-specific morphological probability effects of bi-dialectals in both of the comprehension and production borrowing tests. In the comprehension borrowing, both groups of bi-dialectals showed different similarity effects for SH-more-probable vs. SH-less-probable targets, both differing significantly from the faciliatory effect of monolectals of cross-dialectal similarity. These findings suggest that bi-dialectals, even when recognizing cross-dialectal loanwords in an unfamiliar dialect, tend to create lexical representations for the loanwords by re-encoding etymologically related morphemes between the two dialects, while monolectals do not re-encode compound constituents.

Evidence for the cross-dialectal morpheme-based recoding of bi-dialectals was also found in production borrowing. However, only the SC-SH bi-dialectals were more effective and efficient in creating SH-more-probable nonce loanwords than in creating SH-less-probable loanwords. Thus, re-encoding of compound constituents in production borrowing should be attributed to recipient-dialect-specific experience. Unlike in comprehension borrowing, only bi-dialectals who speak the recipient dialect can make use of the ETE morphemes to produce loanwords.

Again, zooming out to the level of a linguistic community, one can imagine that a linguistic community with more bi-dialectals would have more cross-dialectal loanwords that are aligned at morphemic level with the source forms, whereas a linguistic community with more monolectals when in contact with another dialect would be biased toward cross-dialectal loanwords that are phonologically adapted with whole words as the unit of borrowing. This theory may also apply to long-term co-evolving languages given additional conditions (such as biliteracy, supposedly). For instance, since the late-nineteenth-century, after the Japanese colonization of Korea, with the number of Korean-Chinese bilinguals decreasing significantly, the old morpheme-based Chinese loan words in Korean have been lost to a large degree, whereas more recent Chinese loan words in Korean are scarce and mostly holistically casted (Yu and Wu, 2021).

The findings presented in Short-Term Bi-Dialectal Exposure Facilitates Production Borrowing by Enriching Instead of Increasing Lexical Exposure and Bi-Dialectals Store New Lexical Items, while Monolectal Assimilate Lexical Variants sections suggested that language ecology (social bi-dialectism vs. social monolectism), which is important for the evolution of loanwords and dialects in contact, takes effect with deep-rooted cognitive motives. It has been commonly known by contact linguists that the introduction of loan forms begins with multiple alternative forms but only one or two forms would finally be established in the linguistic community (for examples, see Weinreich, 1953; You, 2016). Our findings suggested that the competing loan forms may emerge from different cognitive routes of lexical borrowing, in that bi-dialectals favor compound borrowing based on morphemes, which focuses on the morpheme-level of units as the target of borrowing, while monolectals favor assimilating holistically casted transferred forms, which focuses on the whole-word-level of units as lexical variants of existing native words. We could further infer that the proportions and relative social statuses of bi-dialectals vs. monolectals in the linguistic community may influence the fate of candidate loan forms, wherein a demographically dominant group of speakers may lead the trend by using loan forms that fit their favored route of borrowing. While the collectively favored loan forms got quickly established and turn into social conventions, the alternative forms became obsolete. This inference in real-world language communities may be further implicated in agent-based computational simulation.

### Dialectal Backgrounds Influence Inference but Not Conflict Resolution in Comprehension Borrowing

The comprehension borrowing experiment showed that low-competition context facilitates lexical identification. However, dialectal backgrounds showed no interaction with the high- and low-competition contexts. Thus, bi-dialectals did not show any advantage in conflict resolution in this cross-dialectal lexical borrowing experiment.

However, regarding the noise trials, SC-OD bi-dialectals were less likely to use inference to figure out the association of the image and the auditory input. Since such inference was not mandatory in the experiment, this difference may be due to the selective lack of adherence of these bi-dialectals to the *mutual exclusivity principle* (Markman, 1992) under the cognitive pressure from the context of an unfamiliar dialect. It is known that even monolingual children can accept the violation of MEP as it happens across languages (Frank and Poulin-Dubois, 2002), which may be a similar situation as the SC-OD bi-dialectals encountered in this task.

Our findings and these language-development data taken together suggested that monolingualism or monolectism may not be the default setting of language evolution. Whether adults or children, monolinguals, or bilinguals, we are always ready to neglect the *mutual exclusivity principle* as long as the two-to-one form-meaning association appears across linguistic varieties. This finding suggested that lexical development in highly bilingual communities and the corresponding language co-evolution may be grounded on a different cognitive basis other than *mutual exclusivity*.

In sum, this study tested the roles of long-term (age, bi-dialectism, and recipient-language-specific experience) and short-term (bi-dialectal lexical exposure and target-specific exposure) linguistic experience in comprehension and production borrowing. The results showed that bi-dialectism provides a general protective influence against age-related deterioration of word learning and cross-dialectal lexical borrowing abilities. Bi-dialectal exposure takes effect not only by increasing the amount of lexical exposure, but more importantly by enriching the experience of bi-dialectals with target new words and helping them encode and decode words more deeply. The long-term bi-dialectal experience of individuals, as well as their short-term exposure to each specific loanword, may collectively shape the route of lexical evolution of co-evolving linguistic varieties.

## Data Availability Statement

The datasets presented in this study can be found in online repositories. The names of the repository/repositories and accession number(s) can be found below: The datasets Cross-Dialectal Novel Word Learning and Borrowing for this study can be found in the Open Science Framework https://osf.io/p9yw4/.

## Ethics Statement

The studies involving human participants were reviewed and approved by the IRB Commitee of East China Normal University. The patients/participants provided their written informed consent to participate in this study.

## Author Contributions

JW designed and carried out the experiment, analyzed the data, and drafted the manuscript. WZ suggested several critical points regarding the theoretical background and implications in language evolution. MH suggested the theoretical implication of MEP. NOS revised the manuscript multiple times and improved the logical flow, layout, and phrasing in detail. All authors contributed to the article and approved the submitted version.

## Funding

This work was supported by Chinese Fundamental Research Funds for the Central Universities (2017ECNU-YYJ017), by Shanghai Philosophy and Social Sciences Fund (2017BYY001), and by National Social Science Fund Major Projects (18ZDA296).

## Conflict of Interest

The authors declare that the research was conducted in the absence of any commercial or financial relationships that could be construed as a potential conflict of interest.

## Publisher's Note

All claims expressed in this article are solely those of the authors and do not necessarily represent those of their affiliated organizations, or those of the publisher, the editors and the reviewers. Any product that may be evaluated in this article, or claim that may be made by its manufacturer, is not guaranteed or endorsed by the publisher.

## Footnotes

^1^Alternatively, individuals of a linguistic society can (a) learn foreign words for old concepts that have existing native words, or (b) create new native forms for new concepts without reference to foreign forms (based on Haugen, 1950 and Weinreich, 1953; as reviewed by Grzega, 2003). However, these two types would not influence the source language as much as lexical borrowing.

^2^Here we refer to http://ccdc.fudan.edu.cn/linguae/ltcPhonology.jsp and use *Wuyun Pan*(潘悟云) 's reconstructions of Middle Chinese pronunciation. Some Chinese characters have multiple phonological status in the Middle Chinese rime dictionaries *Guangyun*  and *Jiyun*. Here we use the phonological status  for the reconstruction of “佛”, for “佛”, and (*Guangyun* only) for “菩”.

^3^This fact has long been noticed by Chinese linguists in their research regarding literary *Wen* and colloquial *Bai* lexical variants in Chinese dialects (e.g., You, 2000, pp. 137).

^4^Shanghainese belongs to the *Wu* family of Chinese dialect (Chen and Gussenhoven, 2015). To control for the influence of mutual-intelligibility (Tang and Van Heuven, 2009), SC-OD participants who speak a *Wu* dialect were excluded.

^5^Wu, J. (in preparation). *Natural Text Corpus of Shanghainese*.
