# Supplementary material for: Cross-Dialectal Novel Word Learning and Borrowing
Source: Front Psychol. 2021 Sep 30;12:734527. doi: 10.3389/fpsyg.2021.734527 (PMC8515950; doi:10.3389/fpsyg.2021.734527)
Supplement: Supplementary file 1 [file Data_Sheet_1.ZIP › Appendix1 Procedure for selection of nonce ETE pairs.docx]

Appendix 1 Procedure for selection of nonce ETE pairs

(1) Two sets of candidate mono-syllabic morphemes (represented with characters) were extracted from an SH natural text corpus and a comparable SC text corpus (both written in Chinese characters, built by the author, containing 0.2 million words, Wu, 2019)^[[1]](#endnote-1)^, based on character frequencies of the morphemes in SH and SC. As shown in the upper panels of Figure 2, the two sets both have median frequencies in SC but differ in SH frequency (**SH-less-probable v.s. SH-more probable)**. The suitable SH-more-probable morphemes naturally carry more concrete meanings than the SH-less-probable morphemes.

(2) Morphemes with extremely low position-related morphological probability were excluded from the two sets.

(3) The candidate morphemes were then crossed within sets to form SH-more/less-probable **candidates of disyllabic nonce ETE pairs**, as shown in the middle panel of Figure 2.

(4) These pairs were screened to excluded existing words and meaningful phrases, yielding 530 **screened** **candidates**.

(5) Since SH has two parallel sets of rules for tone Sandhi (Duanmu, 1999; Jie Zhang & Yuanliang Meng, 2012) and rich pronunciation variants (You, 2013), four young SH speakers (also SC-SH bi-dialectals) from *Yangpu*, *Hongkou*, *Minhang*, and *Pudong* Districts of Shanghai read them in SH for recordings. The author selected candidates which were pronounced consistently by at least three of the four speakers. This procedure finally

**Reference:**

Duanmu, S. (1999). Metrical structure and tone: evidence from Mandarin and Shanghai. *Journal of East Asian Linguistics*, *8*, 1–38.

Jie Zhang, & Yuanliang Meng. (2012). *Structure-Dependent Tone Sandhi in Real and Nonce Words in Shanghai Wu*.

Wu, J. (2019). *Natural text corpus of Shanghainese (in progress)*.

You, R. (Ed.). (2013). *A linguistic survey of Shanghai dialects (上海地区方言调查研究)* (1st ed.). Shanghai: Fudan University Press.

1. No alternative SH natural text corpus and frequency data was available before this research. [↑](#endnote-ref-1)
